# Physicochemical properties and molecular mechanisms of different resistant starch subtypes in rice

**DOI:** 10.3389/fpls.2023.1313640

**Published:** 2024-01-08

**Authors:** Cheng Liang, Haoyang Xu, Hui You, Ouling Zhang, Yiman Han, Qingyu Li, Yungao Hu, Xunchao Xiang

**Affiliations:** ^1^ Lab of Plant Molecular Genetics and Breeding, Southwest University of Science and Technology, Mianyang, China; ^2^ Rice Research Institute, Southwest University of Science and Technology, Sichuan, Mianyang, China; ^3^ School of Medicine, Tsinghua University, Beijing, China

**Keywords:** rice (*Oryza sativa* L.), resistant starch, gene expression patterns, targeted-gene association study, gene interaction, starch granule properties

## Abstract

Resistant starch (RS) can help prevent diabetes and decrease calorie intake and that from plants are the main source of mankind consumption. Rice is many people’s staple food and that with higher RS will help health management. A significantly positive correlation exists between apparent amylose content (AAC) of rice and its RS content. In this study, 72 accessions with moderate or high AAC were selected to explore the regulatory mechanisms and physicochemical properties on different proceeding types of rice RS. RS in raw milled rice (RSm), hot cooked rice (RSc), and retrogradation rice (RSr) showed a wide variation and distinct controlling mechanisms. They were co-regulated by *Waxy* (*Wx*), *soluble starch synthase* (*SS*) *IIb* and *SSI*. Besides that, RSm was also regulated by *SSIIa* and *SSIVb*, RSc by *granule-bound starch synthase* (*GBSS*) *II* and RSr by *GBSSII* and *Pullulanase* (*PUL*). Moreover, *Wx* had significant interactions with *SSIIa*, *SSI*, *SSIIb* and *SSIVb* on RSm, but only the dominant interactions with *SSIIb* and *SSI* on RSc and RSr. *Wx* was the key factor for the formation of RS, especially the RSc and RSr. The genes had the highest expression at 17 days after flowering and were beneficial for RS formation. The longer the chain length of starch, the higher the RS3 content. RSc and RSr were likely to be contained in medium-size starch granules. The findings favor understanding the biosynthesis of different subtypes of RS.

## Introduction

Rice (*Oryza sativa* L.) represents the staple food and provides the body’s primary source of carbohydrates and calorie intake (Butardo et al., 2016). As the high standard of living leads to the frequent occurrence of diabetes, people gradually pursue a low-sugar and low-calorie diet, especially on selecting starchy food. According to digestive qualities, starch is classified as rapidly digestible starch, slowly digested starch, or resistant starch (RS) (Bemiller, 2019). RS has significant effect in preventing diabetes, improving lipid function and helping with weight management (Ma et al., 2018). Based on its properties and botanical origin, RS is divided into five subtypes (RS1 - 5) (Xia et al., 2018). The major RS subtypes, RS2, RS5 and RS3, are respectively present in raw, cooked and cold rice (You et al., 2022). RS2 refers to starch that is structured in a compact radial pattern in the form of native crystalline structures of B and C and is found largely in cereals rich in amylose as ungelatinized resistant granules (Sajilata et al., 2006). RS3 is retrograded starch with a semi-crystalline double helical structure that is produced during the chilling process of cooked rice by amylose and amylopectin (Alhambra et al., 2019). RS5 is complex substance of starch and lipid, which cannot be digested because its presence in starch granules restricts their swelling if being cooked (Zhou et al., 2016).

As a special type of starch, RS was also correlated with physicochemical indicators of starch such as apparent amylose content (AAC), gel consistency (GC), gelatinization temperature (GT) and pasting viscosity parameters (PVPs). RS content was positively correlated with AAC (Kong et al., 2015). The consistency viscosity (CSV), setback viscosity (SBV), and pasting temperature (PaT) of PVP were correlated with RS (Chen et al., 2017). RS2 have a positive correlation with the hot paste viscosity (HPV) and cool paste viscosity (CPV). Meanwhile, the PVPs were strongly negatively correlated to RS3 (Ding et al., 2019). The formation of RS is closely related to the properties of starch granules. The ratio of amylose to amylopectin, as well as the number of long and short branches in amylopectin, had an impact on RS content (Hu et al., 2004). What’s more, the size, shape and granule diameter of starch particles also affect RS content. Rice mutants (*RS111*) with high RS in hot cooked rice (RSc) had primarily round or oval starch grains, whereas R7954 and Zhong9B with high RS in raw milled rice (RSm) had pentagonal and angular starch grains (Yang et al., 2005). Some varieties with high RS had small starch granules (Chia et al., 2017). In addition, RS2 and RS3 were significantly positively correlated with fraction B2 chains (25 < DP < 36) and fraction A chains (DP < 12), respectively. However, RS3 was significantly negatively correlated with granular size, fraction B1 chains (13 < DP < 24) and fraction B3 and longer chains (DP > 37) (Ding et al., 2019). The formation and physicochemical properties of rice starch were regulated by starch synthesis-related genes (SSRGs), and their corresponding enzymes were also involved in the RS synthesis.

Starch biosynthesis is a complicated process involving several enzymes in various metabolic reactions, finally determining the formation and properties of starch granules. RS biosynthesis was determined by interaction of a network of starch biosynthetic enzymes. Mutations in the genes encoding granule-bound starch synthase (GBSS), soluble starch synthase (SS), and starch-branching enzyme (BE) have been reported to influence RS contents in rice, maize, and wheat (Zhou et al., 2016; Shen et al., 2022). GBSSI, encoded by the *Waxy* (*Wx*) gene, is a key enzyme for amylose biosynthesis in the endosperm. In rice, the broad variation of amylose content is due mainly to allelic polymorphism at the *Wx* locus, such as *Wx^a^*, *Wx^b^*, *Wx^op/hp^*, *Wx^mq^*, *Wx^la^*, *Wx^mp^*, *Wx^mv^* and *wx* (Zhang et al., 2019; Zhou et al., 2020). The increased RS in the *Wx^a^* background is mainly due to high AC that promotes the formation of RS3 and RS5 (Shen et al., 2022; You et al., 2022). Genetic analyses showed that a novel allele mutation of *BEIIb* (*sbe3 - rs*) was responsible for the production of high RS in rice (Yang et al., 2016), and defective soluble starch synthase gene (*ssIIIa*) was responsible for RS5 production (Zhou et al., 2016). In maize, a high amylose level was associated with a high RS content. A study found that the RS contents of maize *amylose-extender* (*ae*) mutant lines were larger than that of existing inbred lines (Li et al., 2008). The production of maize *ae* mutant was mainly caused by mutation of *BEIIb* (Stinard et al., 1993). In addition, Pullulanase (PUL) was able to remove the branch of amylopectin and resulted in higher RS content in waxy maize starch. A maize mutation of *SSIII* dramatically increased the amylose content and decreased the amylopectin content and *φ,β*-limit dextrins, suggesting that it may conducive to improving RS (Zhu et al., 2013). The multiple members of SSRGs and rich polymorphisms of a single gene and their interactions complicate the regulatory network of starch synthesis and expand the variation of starch properties in rice. The current research found that the RSm, RSc, and retrogradation rice (RSr) of the same samples differed significantly (You et al., 2022). However, the mechanism of how do the SSRGs and their interactions regulate the different RS subtypes is not yet well understood although several literatures have discussed it. To understand the specific molecular mechanisms and the regulating network for RSm, RSc, and RSr is important since it is in favor of increasing the RS3 and RS5 contents in rice. We hypothesized that the genes that played roles in RS subtypes biosynthesis were regulated by different genetic networks and their expression peaks appeared at certain grain-filling stages, and being beneficial for RS formation. In the study, the variations of RS subtype in raw starch, cooked starch and retrogradation starch and the genotypes in 72 non-glutinous rice accessions with moderate or high AAC (> 20%) and a glutinous rice (control) were investigated, and systematically revealed the regulatory mechanisms for the SSRGs and their interactions on RSm, RSc and RSr by targeted-gene association study (TGAS) and gene interaction analysis et cetera. The results in favor us to understand their relationship and genetic regulation in rice and select excellent varieties with high-RSc or RSr.

## Materials and methods

### Materials

A total of 72 non-glutinous accessions (**Supplementary Table 1**) with moderate or high AAC (>20%) and a glutinous variety ‘XKN149R’ (control) were planted and managed routinely in an experimental field at Southwest University of Science and Technology (Sichuan, China) under natural environment. Three single plants were taken from each accession at the fully mature stage (biological replicates). The seeds were conserved at room temperature for three months to balance the moisture content after dried. The samples were dehulled by dehuller (TR-200; Kett, Tokyo, Japan). The brown rice was polished by polisher (Pearlest; Kett, Tokyo, Japan), and then it was milled to flour in a Laboratory Mill (LM3100; Petern, Malmö, Sweden) using a 100-mesh sieve. All flours were stored in the refrigerator’s cold storage until used.

### Identification of resistant starch and physical-chemical properties

Three different RS (RSm, RSc, and RSr) were measured with the same sample. Cooked rice was produced using the technique described by You et al. (2022). To retrograded rice, a quantity of cooked rice was refrigerated at 4 °C for a week (Zhou et al., 2016). The RS content was measured using the RS Rapid Assay Kit in accordance with the manufacturer’s protocol (Megazyme, Bray, Co. Wicklow, Ireland). Each sample was done twice (parallel test).

The chalkiness rate and chalkiness degree of polished rice were determined by rice appearance scanner (Wan sheng SC - E, Zhejiang). AAC was determined using the spectrophotometry method at 620 nm of colorimetric wavelength, according to Chinese Ministry of Agriculture standard NY/T 2639-2014. GC was determined using Chinese national standards GB/T 17891-1999 in duplicate, with the third determining if the difference of second value exceeded 7 mm. GT was determined using differential scanning calorimetry (DSC, Perkin Elmer, Norwalk, CT, USA) by the method of You et al. (2022). To measure the percentage of retrogradation (R%) and retrogradation temperature (TP_r_), the gelatinized starch was stored at 4 °C for seven days, and then used the same thermal program as the GT measurement. TPr was the peak temperature for the retrograded samples. R% was computed using the following formula: R% = △Hr (enthalpy of retrogradation)/△Hg (enthalpy of gelatinization) *100%. Each thermal parameter was determined twice for each sample (parallel test).

PVPs were described using the profile characteristics of the rapid visco analyzer (RVA), which were determined using an RVA (Model No. RVA4500, NewPortSci. Co. Warriewood, Australia) in accordance with the manufacturer’s instructions and the American Association of Cereal Chemists’ standard method: AACC61 - 02. Breakdown viscosity (BDV) = PKV - HPV, CSV = CPV - HPV, and SBV = CPV - PKV. Additionally, PaT and peak time (PeT) were also measured. Each value was determined in duplicate for each sample (parallel test).

### DNA extraction and genotyping

The genomic DNAs of 73 cultivars were extracted using the cetyltrimethylammonium bromide technique from fresh leaves (Xiang et al., 2007). 18 SSRGs were selected as target genes, including *ADP-glucose pyrophosphorylase* (*AGP*) *large subunit* (*AGPlar*), *AGP small subunit* (*AGPsma*), *AGP large subunit isoform* (*AGPiso*), *Wx*, *GBSSII*, *BE1*, *BEIIb*, *BEIIa*, *pullulanase* (*PUL*), *Isoamylase* (*ISA1*), *soluble starch synthase* (*SS*)*I*, *SSIIa*, *SSIIb*, *SSIIc*, *SSIIIa*, *SSIIIb*, *SSIVa*, and *SSIVb*. The primers were designed by Tian et al. (2010) and Yan et al. (2010) (**Supplementary Table 2**). At the same time, 48 pairs markers of simple sequence repeats (SSR) (**Supplementary Table 3**) distributed on 12 chromosomes of the rice genome were used for population structure analysis, which eliminated the influence of the population structure on the TGAS and prevented “false linkage”. The method of polymerase chain reactions (PCR) and digestion of the products were consistent with (You et al., 2022).

### Quantitative real-time PCR

Total RNA was respectively extracted from fresh seeds being filling (their husks were removed) at 7, 12, 17, and 22 days after flowering (DAF) using RNAprep Pure Plant Kit (Tiangen, Beijing, China). The qRT - PCRs were performed on a CFX Connect Real-Time PCR Detection System (Bio-Rad) using SsoAdvancedTM Universal SYBR Green Supermix (Xinghan, Shanghai, China). The rice *Actin* gene was used as internal reference. The gene-specific primers of qRT - PCRs were listed in **Supplementary Table 4**. Each experiment had three biological replicates.

### Determination of the morphology and particle size distribution of starch granules

Rice starch particles were separated according to the describing of Peng et al. (1999), and the obtained starch particles were stored at -20 °C. The scanning electron microscope (SEM) (UItra55, Carl Zeiss NTS GmbH, Oberkochen, Germany) was used to visualize the shapes of starch particles. 200 mg starch particles added 40 mL of distilled water, and the samples were sufficiently dispersed by ultrasonic treatment for 5 min. The particle size distribution of samples was then determined using a laser diffraction particle size analyzer (Model LS 13320, Beckman Coulter, USA), which used the instrument’s software to automatically compute granule size.

### Statistical analysis

Pearson’s correlation coefficient, one-way analysis of variance (ANOVA), and multiple comparisons at the Duncan level of *P* < 0.05 and *P* < 0.01 were performed using IBM Statistical Product and Service Solutions software version 22 (SPSS, https://www.ibm.com/analytics/cn/zh/technology/spss/). Cluster analysis, population structure analysis, TGAS and gene interaction analysis were finished according to the method of Zhang et al. (2021). The DPS 9.5 was used to do a cluster analysis of the population based on RS content under various treatments (Zhang et al., 2021). The STRUCTURE 2.3.4 software was used to evaluate the population structure of 73 accessions using the Bayesian clustering method. TGAS was conducted according to the genetic differences identified from 48 molecular markers of SSRGs and mean values of the RS content in 73 rice accessions with three treatments. According to the mixed linear model (MLM) in the TASSEL 5.0 software, the K (the kinship matrix) and Q (population structure matrix) model were adopted to eliminate false positives in TGAS, and the *P*-value (marker) (*P <* 0.05) was used to detect the marker-trait association. The K matrix and Q matrix were respectively calculated by TASSEL 5.0 and STRUCTURE 5.0 software (Wang et al., 2019). The R software’s aov function was used to test the significance of gene interaction (*P* < 0.05) and multi-factor variance analysis (https://www.r-project.org/).

## Results

### Phenotypic variability of resistant starch content and cluster analysis of 73 accessions

**Figure 1:**
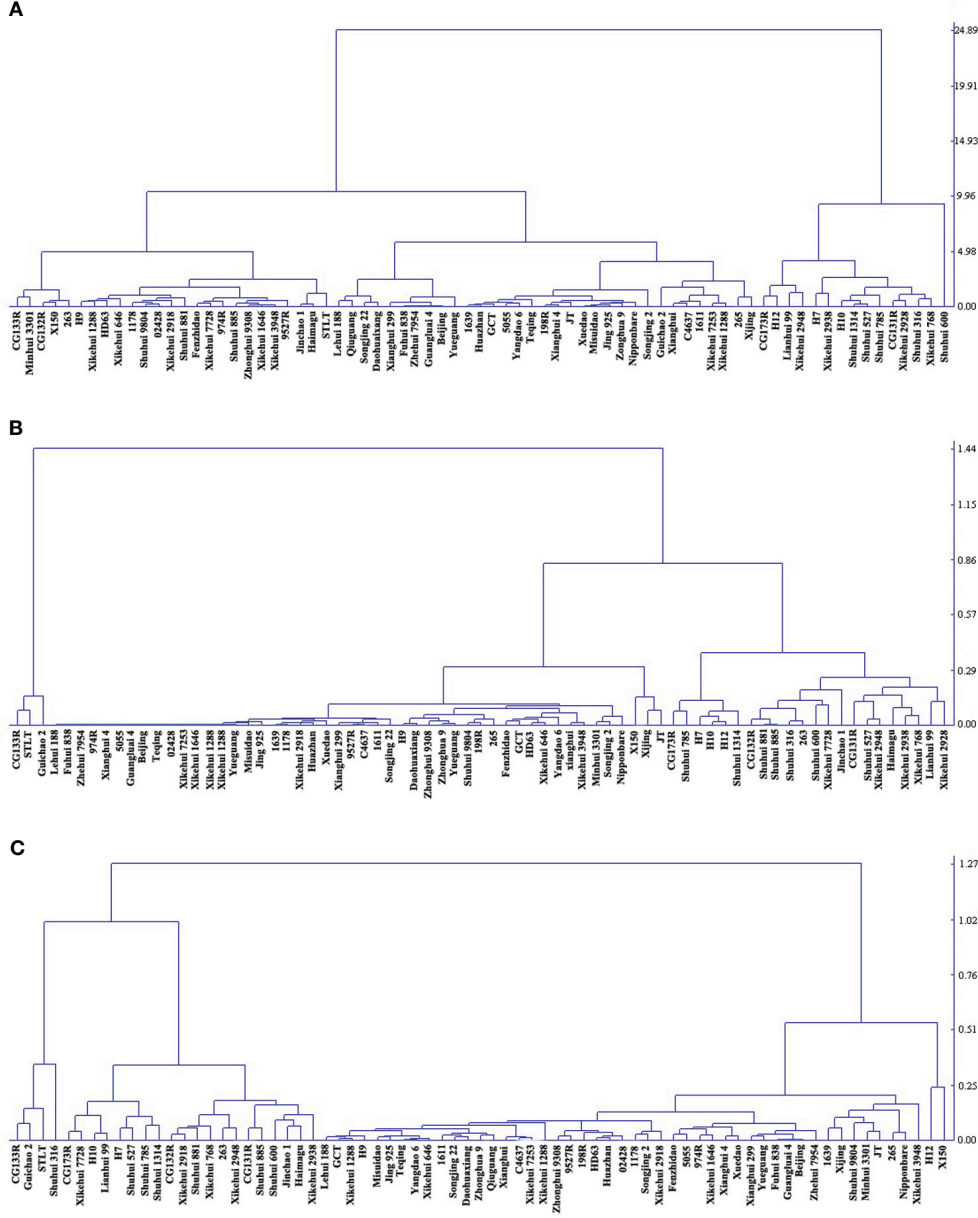
Cluster analysis of based on RS content in three status. **(A)** RSm, resistant starch content in milled sample; **(B)** RSc, resistant starch content in cooked rice; **(C)** RSr, resistant starch content in retrograded rice. The distribution of varieties with resistant starch content from low to high is from left to right on the horizontal axis.

There was extensive variation in RSm from 73 accessions, ranging from 0.18 to 24.82%, and the coefficient of variation (CV) was 73.51%. However, the CV of RSc and RSr were higher than RSm (82.61% and 77.78%, respectively) (**Supplementary Table 5**). The RS contents of 73 accessions were clustered into three categories: high, medium and low by DPS 9.5, and RSm, RSc and RSr had differently clustering results. For RSm, germplasm with high RS (17.51% to 24.82%) accounted for 20.55%, while that with medium (7.33% to 11.65%) and low (0.18 to 6.62%) accounted for 79.45% (**Figure 1A**). The RS content of all germplasm decreased significantly after cooked (*P <* 0.01) (**Table 1**), and only Guichao2, STLT and CG133R were clustered as high RSc (0.97% to 1.07%) in the dendrogram of RSc (**Figure 1B**). After 7 days of retrogradation, RSr content of some germplasm increased slightly, and 4 germplasm (Guichao2, STLT, CG133R and Shuhui316) were classified as high RSr (1.21% to 1.37%) (**Figure 1C**). The decrease of RS content after cooked (RSa, RSa = RSm - RSc) and the increase of RS content after 7 days of retrogradation (RSb, RSb = RSr – RSc) in 73 accessions ranged from 0.17 to 24.14% and 0.01 to 0.62%, respectively, and both of CV exceeded 73.11% (**Table 1**).

**Table 1:** Variation of resistant starch content in different process statuses for 73 accessions.

Types	Min	Max	Mean ± SE	CV (%)
RSm (%)	0.18	24.82	8.87 ± 6.52aA	73.51
RSc (%)	0.01	1.07	0.23 ± 0.19bB	82.61
RSr (%)	0.02	1.37	0.36 ± 0.28bB	77.78
RSa (%)	0.17	24.14	8.63 ± 6.31aA	73.11
RSb (%)	0.01	0.62	0.13 ± 0.11bB	84.61

RSm, RSc and RSr denote RS in raw milled rice, in cooked rice, and in retrograded rice, respectively. RSa is equal to RSm - RSc; RSb is equal to RSr - RSc. Above the column, lowercase and capital letters represent significance at the 0.05 and 0.01 levels, respectively.

### Analysis on the correlation between resistant starch and its physicochemical qualities in 73 accessions

Among the quality parameters of 73 germplasm, most indexes showed moderate or high variation except for GT, ΔHg and TP_r_ (**Table 2**). Significant correlations were found between RS content of different types and multiple quality indexes (**Table 3**). There was a significantly positive correlation among RS content of different subtypes with AAC, HPV, CPV, SBV, CSV (*P <* 0.01). At the same time, GC, TP_r_ and BDV were significantly negatively correlated with RSm, RSc and RSr (*P <* 0.01), respectively. RSm and RSa were significantly positively correlated with GT, but RSc and RSr were significantly negatively correlated with it. RSm and RSa showed a significant correlation with all thermal parameters and RVA profile characteristics (*P <* 0.01) except for ΔHg. RSc was significantly negatively correlated with ΔHg and TP_r_ (*P <* 0.01), while it was positively correlated with the RVA profile characteristics excepted for PKV and BDV (*P <* 0.01), and RSr with the same results. Only RSc had a significant positive correlation (*P <* 0.01) with the chalkiness rate.

**Table 2:** The starch properties of 73 rice accessions.

Traits	Min	Max	Mean	CV%
Chalkiness rate (%)	8.96	100.00	52.88	47.87
Chalkiness degree (%)	1.99	65.34	23.37	63.93
Apparent amylose content (%)	0.00	37.76	21.76	47.93
Gel consistency (mm)	18.00	119.50	58.30	48.52
Gelatinization temperature ( °C)	65.28	79.91	72.82	6.20
Gelatinization enthalpy (J/g)	6.58	11.35	9.02	13.74
Retrogradation temperature ( °C)	47.51	55.05	51.80	3.31
Enthalpy of retrogradation (J/g)	0.17	5.45	2.82	49.73
Percent of retrogradation (%)	1.74	48.89	30.20	40.04
Peak viscosity (cp)	1115.00	3850.00	3207.64	15.20
Hot paste viscosity (cp)	310.50	3240.50	1895.17	29.21
Cool paste viscosity (cp)	433.50	5104.50	3110.66	30.31
Breakdown value (cp)	421.50	23050.00	1312.47	41.32
Setback value (cp)	-1509.00	1482.50	-97.13	75.24
Consistence value (cp)	123.00	2056.50	1215.49	36.73

**Table 3:** Correlations between resistant starch and physicochemical properties in 73 rice accessions.

Trait	RSm	RSc	RSr	RSa	RSb
Chalkiness rate (%)	0.21	0.25*	0.23	0.20	0.12
Chalkiness degree (%)	0.15	0.20	0.18	0.14	0.10
Apparent amylose content (%)	0.73**	0.88**	0.89**	0.71**	0.71**
Gel consistency (mm)	-0.73**	-0.78**	-0.80**	-0.71**	-0.66**
Gelatinization temperature ( °C)	0.36**	-0.36**	-0.27*	0.38**	-0.01
Gelatinization enthalpy (J/g)	0.05	-0.56**	-0.50**	0.08	-0.22
Retrogradation temperature ( °C)	-0.35**	-0.83**	-0.80**	-0.32**	-0.50**
Enthalpy of retrogradation (J/g)	0.40**	-0.20	-0.13	0.42**	0.06
Percent of retrogradation (%)	0.51**	-0.55	0.01	0.53**	0.16
Peak viscosity (cp)	0.32**	0.04	0.05	0.33**	0.08
Hot paste viscosity (cp)	0.52**	0.70**	0.68**	0.50**	0.49**
Cool paste viscosity (cp)	0.68**	0.77**	0.77**	0.66**	0.61**
Breakdown value (cp)	-0.24*	-0.68**	-0.65**	-0.22	-0.43**
Setback value (cp)	0.56**	0.83**	0.82**	0.54**	0.62**
Consistence value (cp)	0.79**	0.76**	0.79**	0.78**	0.67**

RSm, RSc and RSr denote RS in raw milled rice, cooked rice, and retrograded rice, respectively. RSa equals RSm minus RSc; RSb equals RSr minus RSc. * and ** denote significance when P < 0.05 (two - tail) or P < 0.01 (two - tail).

### Analysis on population structure and genetic relationship

159 amplified polymorphic bands were detected in 73 accessions using 42 pairs of SSR primers, and there were 3.786 alleles for each locus averaged, which ranged from 2 to 8 (RM333) (**Supplementary Table 5**). The average value of polymorphism information content (PIC) was 0.474, ranging from 0.027 (RM308) to 0.751 (RM335). By and large, the bigger the PIC, the more genetic difference of the materials (Zhang et al., 2021). In the 73 accessions, it included 21 highly polymorphic sites (PIC > 0.5) and 17 moderately polymorphic sites (0.25 < PIC < 0.5). The results showed that there was richly genetic polymorphism in the 73 accessions, which could be used for subsequent analysis on population structure.

The model-based population structure was analyzed among the 73 rice accessions so that a significant population structure was provided. The Mean L (k), the mean of log-likelihood values, grew as the model parameter K increased, which had no significant inflexion point in the line plot so that cannot be used for population division (**Supplementary Figure 1A**). Zhang et al. (2021) used ΔK as the diagnostic criterion for determining an appropriate K value, and so got the greatest K value at K = 4 (**Supplementary Figure 1B**). According to the Q values of each material at K = 4 (**Supplementary Table 6**), 73 materials were divided into four groups (**Supplementary Figure 1C**), which were consistent with the result of the neighbour-joining tree based on Nei’s genetic distances (**Supplementary Figure 2**). Furthermore, most of the cultivars in the four subpopulations had a relatively pure genetic background (only one color), and a few materials had two or more background colors, which might be related to the hybridization of the ancestral subpopulations. The heat map of the kinship was drawn based on the kinship matrix (**Supplementary Figure 3**). At the same time, the fixed effect matrix was determined to prepare for the TGAS by combining the Q matrix at K = 4.

### Targeted-gene association study on different resistant starch subtypes

**Figure 2:**
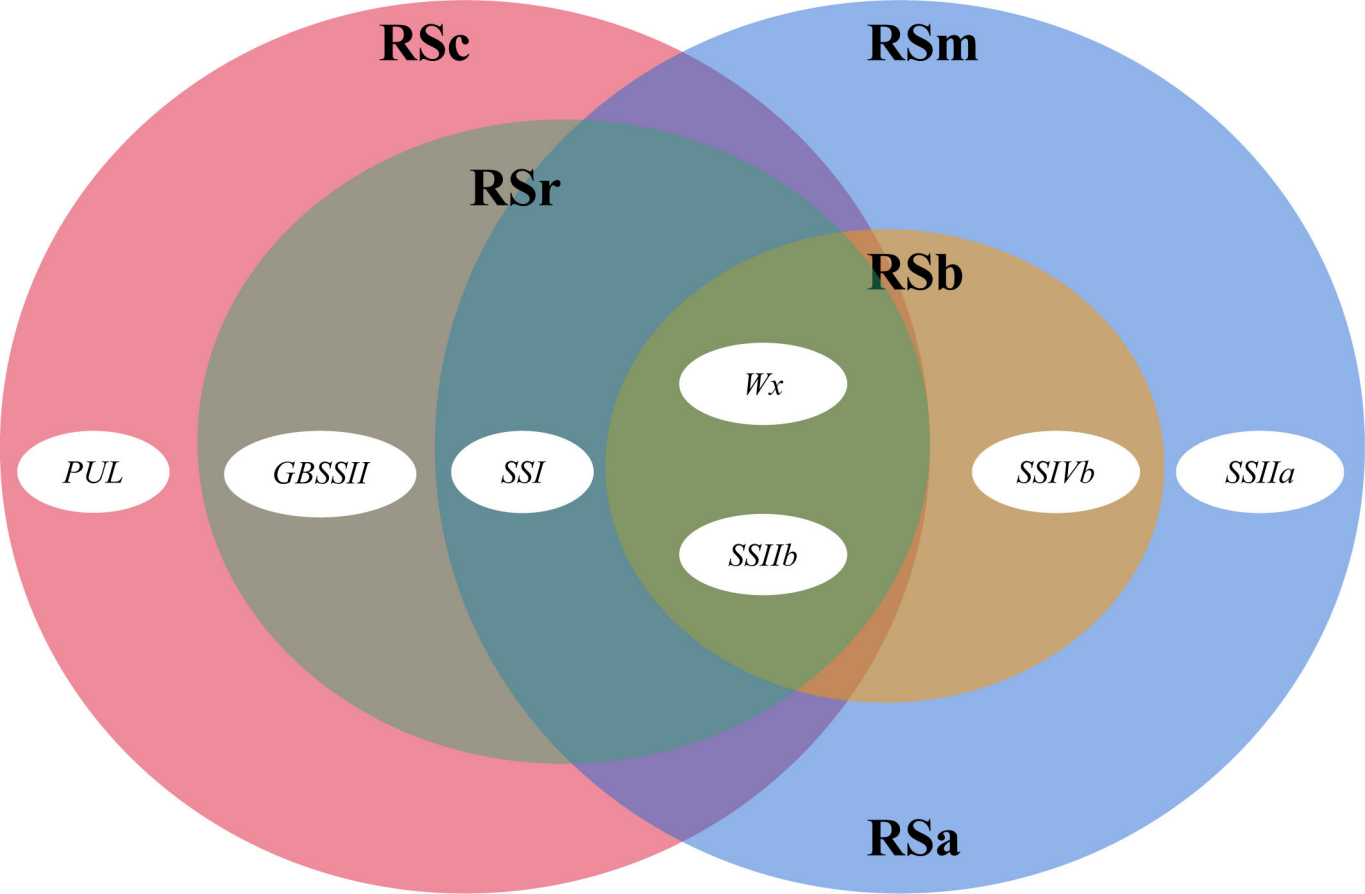
An overview of the genes involved in rice RS. The four big ovals show the resistant starch content of milled rice (RSm, blue), cooked rice (RSc, pink), retrograded rice (RSr, brown), RSa is equal to RSm – RSc (blue), and RSb is equal to RSr – RSc (yellow), while the little ovals reflect the role of each gene.

TGAS was conducted based on the genotypes of 18 SSRGs (**Supplementary Table 7**), phenotypic data, population structure and genetic relationship. The results showed that the synthesis of rice RS was controlled by multiple genes (**Table 4**). *Wx*, *SSIIb*, *SSIVb*, *SSIIa* and *SSI* had significant genetic effects on the RSm (*P <* 0.01). Compared with RSm, *GBSSII* and *PUL* significantly affected RSc (*P <* 0.05), but *SSIVb* and *SSIIa* had no significant genetic effects on it. In addition, *Wx*, *SSI*, *SSIIb* and *GBSSII* were involved in the genetic regulation of RSr (*P <* 0.01). *Wx* had the most significant genetic effect on RS in the three processing statuses than other SSRGs (**Figure 2**). RS2, as the principal component of RSa and RSm, had the same regulating genes as RSm. RS3, as the main component of RSb, was regulated by *Wx*, *SSIIb* and *SSIVb* (*P <* 0.01), and their genetic effect decreased successively.

**Table 4:** Targeted-gene association study of resistant starch in different processing statuses.

Trait	Gene	*F* - Value	*P* - Value	Trait	Gene	*F* - Value	*P* - Value
RSm	*Wx*	11.869	4.22E-05	RSa	*Wx*	11.085	7.50E-05
	*SSIIb*	11.279	0.001		*SSIIb*	11.062	0.001
	*SSIVb*	8.313	6.25E-04		*SSIVb*	8.270	6.47E-04
	*SSIIa*	7.458	0.001		*SSIIa*	8.264	6.50E-04
	*SSI*	6.016	0.017		*SSI*	5.670	0.020
RSc	*Wx*	14.062	8.93E-06	RSb	*Wx*	10.319	1.33E-04
	*SSI*	7.881	0.007		*SSIIb*	4.974	0.029
	*GBSSII*	5.564	0.021		*SSIVb*	3.267	0.045
	*SSIIb*	4.804	0.032				
	*PUL*	4.209	0.044				
RSr	*Wx*	15.361	3.68E-06				
	*SSI*	6.703	0.012				
	*SSIIb*	5.628	0.021				
	*GBSSII*	4.630	0.035				

RSm, RSc and RSr denote RS in raw milled rice, cooked rice, and retrograded rice, respectively. RSa is equal to RSm - RSc; RSb is equal to RSr - RSc.

### Gene interaction analysis on different resistant starch subtypes

**Figure 3:**
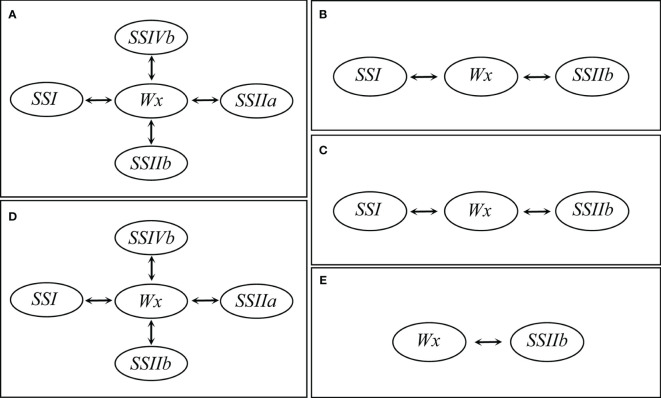
The gene interaction effects on resistant starch at the different processing statuses. **(A)** RSm, resistant starch content in milled rice; **(B)** RSc, resistant starch content in cooked rice; **(C)** RSr, resistant starch content in retrograded rice; **(D)** RSa is equal to RSm – RSc; **(E)** RSb is equal to RSr – RSc.

The effects of SSRGs interactions on RS content extensively existed in different subtypes of RS. The interaction between *Wx* and *SSIIb* significantly affected RSm, RSc, RSr, RSa and RSb (**Table 5**). As the regulatory center of interaction effects, the interactions of *Wx* with *SSIIa*, *SSIIb*, *SSI* and *SSIVb* significantly regulated the biosynthesis of both RSm and RSa (**Figures 3A, D**). Moreover, *Wx* also showed significant interactions with *SSI* and *SSIIb* in regulating both the RSc and RSr (**Figures 3B, C**). Only the interaction of *SSIIb* and *Wx* affected the RSb (**Figure 3E**). The above results showed that the biosynthesis of RS in rice was also regulated by the interactions of related genes, especially the interaction effects of *Wx* with other SSRGs.

**Table 5:** The interaction effects of starch synthesis-related genes.

Trait	Gene	*F* - Value	*P* - Value	Trait	Gene	*F* - Value	*P* - Value
RSm	*Wx×SSI*	6.596	0.013	RSa	*Wx×SSI*	6.087	0.016
	*Wx×SSIVb*	3.390	0.040		*Wx×SSIVb*	3.394	0.040
	*Wx×SSIIb*	8.285	0.005		*Wx×SSIIb*	7.656	0.007
	*Wx×SSIIa*	32.153	0.000		*Wx×SSIIa*	36.222	0.000
RSc	*Wx×SSI*	6.602	0.012	RSb	*Wx×SSIIb*	4.409	0.040
	*Wx×SSIIb*	7.976	0.006				
RSr	*Wx×SSI*	7.400	0.008				
	*Wx×SSIIb*	9.649	0.003				

RSm, RSc and RSr denote RS in raw milled rice, in cooked rice, and in retrograded rice, respectively. RSa is equal to RSm - RSc; RSb is equal to RSr - RSc.

### Expression level analysis of key genes and their correlation with resistant starch

**Figure 4:**
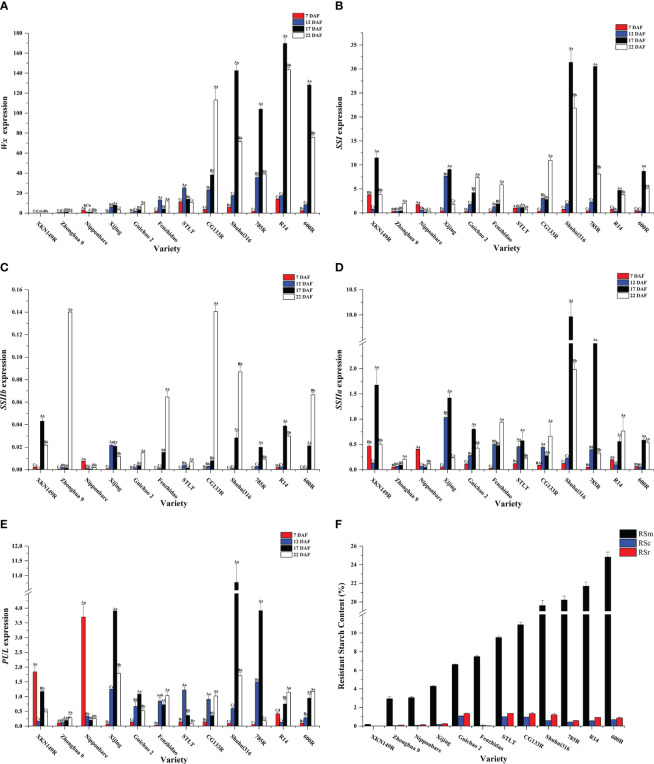
Gene expression analysis of different cultivars at different stages of postanthesis development. **(A)** *Wx*, *Wxay*; **(B)** *SSI*, *soluble starch synthases I*; **(C)** *SSIIb*, *soluble starch synthases IIb*; **(D)** *SSIIa*, *soluble starch synthases IIa*; **(E)** *PUL*, *pullulanase*; **(F)** The accessions for gene expression analysis and their RS contents. RSm, RSc and RSr denote RS in raw milled rice, in cooked rice, and in retrograded rice, respectively. Letters above column indicate ANOVA of gene expression of the same cultivar at different postfloral stages. Lowercase and capital letters denote significant at 0.05 and 0.01 levels, respectively.

**Figure 5:**
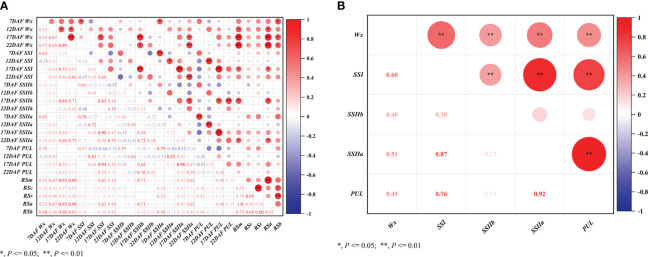
Correlation analysis of gene expression. **(A)** Correlations between relative expression of the same gene at different stages, correlations between the relative expression of different genes at the same stage, and correlations between the relative expression of different genes at different stages and different subtypes of resistant starch. RSm, RSc and RSr denote RS in raw milled rice, in cooked rice, and in retrograded rice, respectively. RSa equals to RSm - RSc; RSb equals to RSr – RSc; **(B)** Pearson all pairwise correlations between relative expression of different genes at different postfloral stages.

According to the cluster analysis results of the RSm, RSc, and RSr, twelve accessions were selected out for the analysis of expression level of key genes at different developmental stages (**Figure 4F**). *Wx* had higher expression levels in the endosperm comparable to the other genes (**Figure 4A**)*. SSIIa* and *PUL* had similar expression levels and were slightly lower than *SSI* (**Figures 4B, D, E**), while *SSIIb* was barely expressed in the endosperm (**Figure 4C**). The expression levels of these genes in germplasm was firstly increased and then decreased along with the developing of fresh seeds, and reaching a peak between 17 DAF and 22 DAF. As for glutinous ‘XKN149R’, however, the *Wx* gene was mutated as recessive *wx* gene because of a 23 bp insertion at the second exon of *Wx*. The *wx* was not expressed (**Figure 4A**) even though other genes had higher expression levels at different developmental stages, especially at 17 DAF, and its RSm, RSc and RSr were all lowest than that of other accessions (0.18%, 0.012% and 0.018%, respectively). It indicated that *Wx* was the key factor for the formation of RS, especially the RSc and RSr. In addition, RSm and RSa were significantly positively correlated not only with the expression of *Wx* at 12 DAF, 17 DAF and 22 DAF, but also with the expressions of *SSI*, *SSIIa* and *SSIIb* at 17 DAF (*P <* 0.05 or *P <* 0.01) (**Figure 5A**). While the RSb was only significantly positively correlated with the expression of *Wx* at 7 DAF, 17 DAF and 22 DAF. These results further confirmed the regulatory role of key genes on different subtypes of RS in the TGAS. Except for *PUL*, the expression of other genes at 17 DAF was significantly positively associated with the expression of *Wx* (*P <* 0.05 or *P <* 0.01), which was consistent with the results of gene interactions, and thus it appeared likely that the interactions between *Wx* and other genes would elevate their transcriptional level. In addition, all the three genes, *SSI*, *SSIIa* and *PUL*, were significantly and positively correlated with each other in terms of expression level (*P <* 0.05 or *P <* 0.01) (**Figure 5B**), and indicating a co-regulatory relationship among the three genes in amylopectin synthesis.

### Analysis on morphology and granularity distribution of starch particles

**Figure 6:**
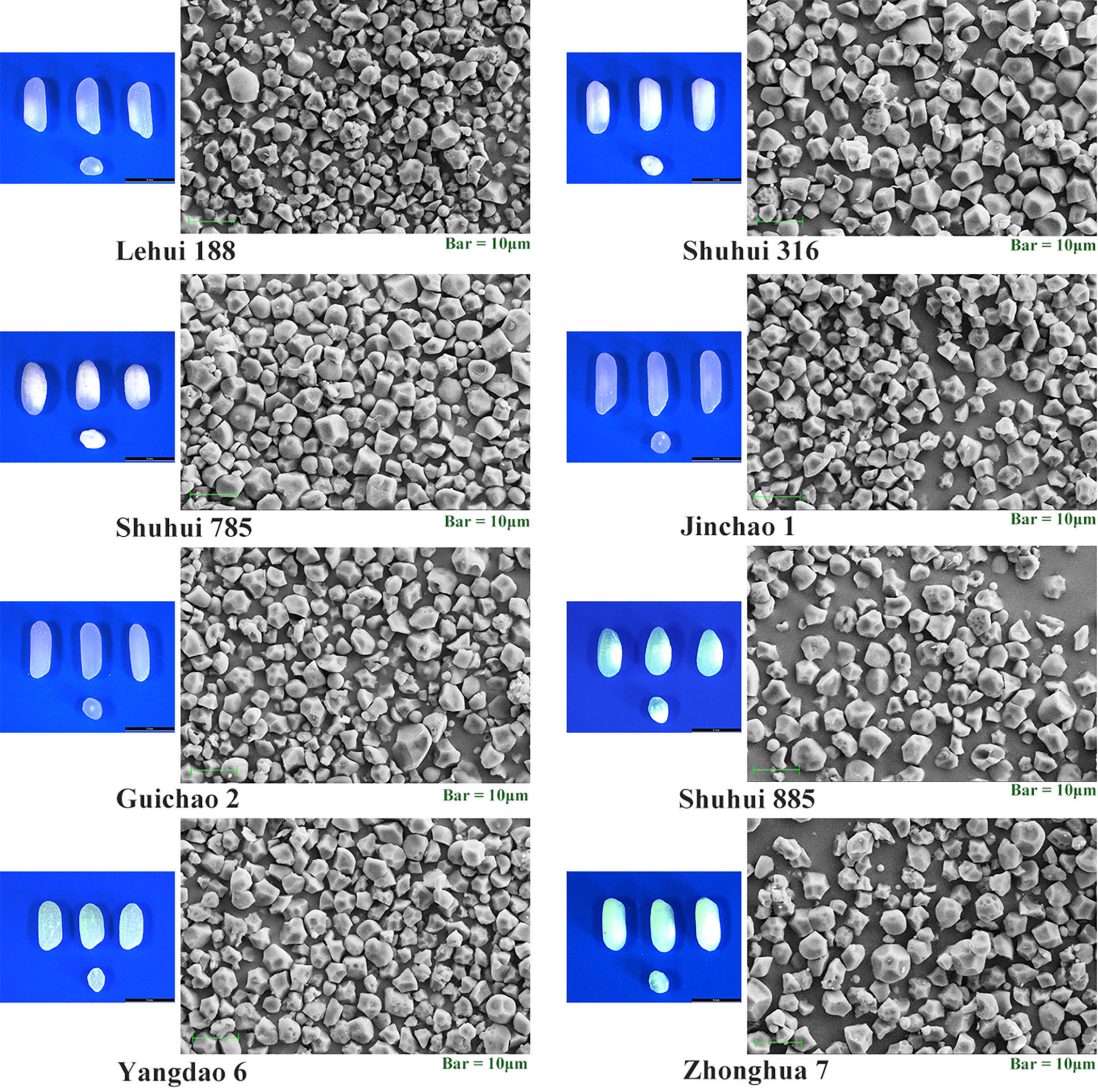
Morphology of seeds and their starch granules for the eight accessions. Illustrative samples of intact seeds and transverse sections for every accession taked by light microscopy are shown on the left. Scanning electron micrographs of starch granules for every accession are shown on the right.

According to the results of cluster analysis, eight accessions with different RS contents (**Table 6**) were selected from 73 accessions to scan the starch particles, and the SEM results showed the size and shape of starch particles in each cultivar differed (**Figure 6**). Most of the starch grains in the eight accessions were pentagonal and angular, and a few were spherical or oval. The results of particle size analysis revealed that their starch particles had a wide distribution, with an average particle size ranging from 4.41 μm to 7.65 μm, and there were significant differences (*P <* 0.01). Furthermore, most of the accessions belonged to medium starch particles (4 μm ≤ particle size *≤* 8 μm) (**Table 7**). The correlation analysis showed that there was no significant correlation between RS contents of different subtypes and average grain size. However, RSc, RSr and RSb were significantly positively correlated with medium grain size starch grains (*P <* 0.05) (**Table 8**), and it demonstrated that medium-sized starch grains could affect RSc and RSr.

**Table 6:** The resistant starch contents of 8 rice accessions.

Accession	Subtype				
	RSm (%)	RSc (%)	RSr (%)	RSa (%)	RSb (%)
Lehui 188	1.88 ± 0.21dD	0.00 ± 0.00 dD	0.08 ± 0.01cD	1.88 ± 0.21dE	0.08 ± 0.01bB
Shuhui 316	19.61 ± 0.81aA	0.59 ± 0.01 bcBC	1.21 ± 0.17 aAB	19.02 ± 0.82aA	0.62 ± 0.16aA
Shuhui 785	20.22 ± 0.59aA	0.41 ± 0.08 cC	0.60 ± 0.02bC	19.81 ± 0.67aA	0.19 ± 0.11bB
Jinchao 1	9.36 ± 0.86bB	0.72 ± 0.08bB	0.86 ± 0.06bBC	8.65 ± 0.78bB	0.14 ± 0.01bB
Guichao 2	6.62 ± 0.00cC	1.08 ± 0.05aA	1.35 ± 0.08aA	5.55 ± 0.05cCD	0.27 ± 0.03bAB
Shuhui 885	7.85 ± 0.11bcBC	0.64 ± 0.02 bBC	0.88 ± 0.06bBC	7.21 ± 0.13bcBC	0.24 ± 0.09bAB
Yangdao 6	3.63 ± 0.24 dD	0.09 ± 0.05 dD	0.07 ± 0.01cD	3.55 ± 0.19dDE	0.02 ± 0.01bB
Zhonghua 9	2.94 ± 0.32 dD	0.06 ± 0.01dD	0.12 ± 0.01cD	2.88 ± 0.33dE	0.06 ± 0.00bB

RSm, resistant starch content in milled sample; RSc, resistant starch content in cooked rice; RSr, resistant starch content in retrograded rice. RSa= RSm-RSc; RSb= RSr-RSc. Lowercases and capital letters indicate significance at P ≤ 0.05 (two-tail) and P ≤ 0.01 (two-tail), respectively.

**Table 7:** Difference of particle size distribution of starch granule for different varieties.

Accession	Mean diameter (μm)	Starch granule distribution			
		Volume percentage (%)			
		0 - 2 μm	2 - 4 μm	4 - 8 μm	> 8 μm
Lehui 188	4.41 ± 0.32deC	17.63 ± 0.12bB	36.72 ± 1.23aA	38.12 ± 0.74cB	7.52 ± 2.00eE
Shuhui 316	6.40 ± 0.06bcAB	19.40 ± 0.07aA	15.06 ± 0.08dD	39.86 ± 0.34bAB	25.68 ± 0.48aA
Shuhui 785	5.60 ± 0.03cdBC	19.33 ± 0.35aA	22.09 ± 0.13cC	40.07 ± 0.31abAB	18.52 ± 0.53bcBC
Jinchao 1	5.02 ± 0.10deC	14.61 ± 0.09dD	34.41 ± 0.49aA	41.03 ± 0.33abAB	9.97 ± 0.71deDE
Guichao 2	4.97 ± 0.03deC	16.11 ± 0.04cC	27.54 ± 0.07bB	42.14 ± 0.41aA	14.21 ± 0.52cdCD
Shuhui 885	7.32 ± 0.38abA	12.86 ± 0.19eE	21.27 ± 0.74cC	39.53 ± 1.22bcAB	26.35 ± 1.76aA
Yangdao 6	7.65 ± 0.39aA	16.37 ± 0.10cC	22.84 ± 0.53cC	38.03 ± 0.73cB	22.77 ± 1.36abAB
Zhonghua 9	6.55 ± 0.26bcAB	19.39 ± 0.02aA	16.53 ± 0.41dD	40.11 ± 0.73abAB	23.98 ± 1.16aAB

Lowercase and uppercase indicate significance at P ≤ 0.05 (two-tail) and P ≤ 0.01 (two-tail), respectively.

**Table 8:** Correlations between resistant starch and starch granule volume.

Subtype	Mean diameter (μm)	Starch granule diameter			
		0 - 2 μm 2 - 4 μm 4 - 8 μm > 8 μm			
RSm (%)	- 0.009	0.344	- 0.399	0.269	0.262
RSc (%)	- 0.222	- 0.423	0.048	0.828*	- 0.066
RSr (%)	- 0.170	- 0.240	- 0.124	0.746*	0.073
RSa (%)	0.002	0.373	- 0.408	0.229	0.271
RSb (%)	- 0.002	0.189	- 0.426	0.342*	0.329

RSm, RSc and RSr denote RS in raw milled rice, cooked rice, and retrograded rice, respectively. RSa is equal to RSm - RSc; RSb is equal to RSr - RSc. * denotes significance when *P* < 0.05 (two - tail).

## Discussion

RS2 and RS5 are respectively the primary subtypes found in raw and cooked rice (Zhou et al., 2016; Ding et al., 2019). Previous studies on recombinant inbred lines (RILs) revealed that RS2 was the main component in 99 rice lines and only had a small amount of RS5 by comparing the RSm (0.03 to 29.42%) and the RSc (0 to 1.71%) (You et al., 2022). Two rice mutants*, be2b* and *be1be2b* with high RS, the RSc was only one-third (3.9%) of RSm (11.6%) for *be2b* mutant and the RSc was three quarters (26.6%) of RSm (35.1%) for *be1be2b* mutant (Miura et al., 2021). RS content of rice was rapidly decreased after cooking, and the RSc was drastically reduced as compared to RSm, but no distinct variation was observed in RSr (You et al., 2022). In this experiment, we made the same conclusion that the RS subtypes of the 73 accessions were mainly RS2, and a small fraction of RS5. At the same time, divergent variability of RSm, RSc and RSr indicated that there were different regulation mechanisms for them. RS2 in rice could not be utilized by the human body because rice was eaten after cooked. Therefore, rice varieties with high RSc and high RSr will better meet the market demand. Fortunately, ‘Guichao2’, ‘STLT’, ‘CG133R’ and ‘Shuhui316’ showed high RSc or RSr in the cluster analysis of rice RS. In particular, Guichao2 belonged to the low category in the clustering of RSm and the high category in both RSc and RSr, which could be used as an excellent resource for development and molecular breeding with high RS.

As a positive correlation existed between the AAC and RS, RSm, RSc, and RSr would increase along with the rise of AAC (You et al., 2022), which is consistent with our research. In addition, the correlation coefficient between AAC and RSc (r = 0.88, *P* < 0.01) was bigger than that between AAC and RSm (r = 0.73, *P* < 0.01) (**Table 3**), which proved that AAC had more significant effect on RSc than RSm (Ding et al., 2019). By investigating the thermal characteristics of rice starch with different subtype RS, it was found that the GT and ΔHg values of *RS111*, a mutant with high RSc, were significantly lower than those of the wild type (Yang et al., 2005). It was consistent with the result that RSc was negatively correlated with GT and ΔHg in our study. As for the rice retrogradation characteristics, present studies have shown that there was a positive correlation between R% and RS3 (Miura et al., 2021), and it indicated that the longer the chain length of starch, the higher the RS3 content. However, R% was also significantly positively correlated with RS2 in this study. In addition, TPr could be used as a predictor of RS content because it was significantly negatively correlated with RSm, RSc, RSr, RSa and RSb (**Table 3**).

SSRGs control structural properties of starch and the fine structure of amylose and amylopectin determines functional properties of rice starch in different ways. Current studies have found that part of SSRGs including *Wx*, *SSIIa*, *BEIIa*, *ISA*, and *AGPsma* regulated RS content in rice by genome-wide association study (GWAS) (Bao et al., 2017; Zhang et al., 2021). A GWAS found that three loci responsible for starch properties were detected on chromosomes 1, 6, and 11, and the most significant SNP corresponded to LOC_Os06g04200 which encoded GBSSI (Praphasanobol et al., 2023). The another GWAS led to the identification of 11 associations for RS on seven chromosomes and five associations for AAC on chromosome 6 (Biselli et al., 2019). Obviously, chromosome 6 of rice was a hot spot for RS regulation. In addition, The additive effect of *SSIIa* together with *Phosphofructokinase* (*pfkB*) explained RS phenotype variation of 19% by TGAS (Parween et al., 2020). However, rice RS is composed of multifarious subtypes, and the synthesis of different subtypes may be regulated by different genes and have different regulatory mechanisms. *Wx^a^* could elevate RSm, RSc and RSr. However, *SSIIa* only played a vital role in regulating RSm (You et al., 2022), which further confirmed that the different RS subtypes were regulated by different genes. In our study, RSm, RSc and RSr have respectively regulatory networks (**Figure 2**), and they all were regulated by *Wx*, *SSI* and *SSIIb*, but each of them was also affected by the other genes individually. It should be emphasized that the significantly genetic effects of *SSI* and *SSIIb* similar to *Wx* have rarely been reported in previous studies on RS genetic mechanism. It might be explained by the fact that down-regulated *SSIIb* expression could decrease AC (Xu et al., 2020), and the AC and ratio of B - type starch granules were negatively affected by *SSI* (Mcmaugh et al., 2014). The regulatory genes of RSm included *SSIIa* and *SSIVb* in our results. *SSIIa* has been identified as a candidate gene for regulating RS (Bao et al., 2017). Its principal role was to control the degree of crystallinity and the amount of fraction A chains in amylopectin (Kong et al., 2015). Rice starch was more resistant to enzymatic digestion when having higher crystallinity, resulting in higher RS content. In wheat and maize, mutations in *SSII* or *SSIII* dramatically increase the AC and change the distribution of amylopectin chain lengths, and suggesting that they may also influence the content of RS (Zhu et al., 2013; Shen et al., 2022). *SSIVb*, as minor gene, was firstly found to regulate RSm, RSa and RSb, which was possibly caused by its ability to alter the morphology and number of starch granules (Macneill et al., 2017). The RS content of the *SSIIIa SSIVb* double mutant is higher than that of the wild type and *SSIIIa*, which suggested the possibility that *SSIVb* might influence the formation of RS (Toyosawa et al., 2016; Shen et al., 2022). The overexpression of *GBSSII* resulted in an increase in the amount of starch and amylose (Jiang et al., 2013), which might explain that *GBSSII* was associated with RSc and RSr. *PUL* affected rice RS content by regulating amylopectin cluster structure (Krishnan et al., 2020), while it was only associated with RSc in this study. PUL could remove the wrongly α-1,6-linked branches, for instance, where the inner chain spacing was such that amylopectin could not form the double helices. Therefore, it could be inferred that *PUL* was involved in regulating amylopectin biosynthesis with a larger molecular weight, which made it more resistant to heat digestion.

Among the multiple allelic variants of *Wx*, *Wx^a^* could effectively increase RSm, RSc and RSr. Moreover the *SSIIa^i^* allele produces higher RS content in *indica* cultivars than does the *SSIIa^j^* allele in *japonica* cultivars (You et al., 2022). Rice accessions carrying deleterious variants in the *SSI* gene have high RS contents (Raja et al., 2017). The latest study found that the loss-of-function *SSIIIb* and *SSIIIa* contributed to the high-RS phenotype (Huang et al., 2023; Wang et al., 2023). However, the function of *SSIVb* to increase the rice RS content needs to be further validated. Starch synthesis is a complex process, which is not only regulated by SSRGs, but also affected by their interactions. The interactions of *Wx*×*PUL*, *ISA*×*PUL*, *SSIIa*×*PUL*, *SSI*×*BEI* and *BEI*×*BEIIb* could explain AC variation of 1.3%, 1.9%, 1.4%, 2.6% and 3.2%, respectively (He et al., 2006). The interaction between *Wx* and *SSIIa* significantly affected rice RSc content through the study of high RS mutant *b10* (Zhou et al., 2016) and meantime had significant genetic effects on RSm, RSr and RSa (*P* < 0.01) (You et al., 2022), which was consistent with our conclusions. The results of gene interactions further prove that the regulatory mechanism of RS2 is more complex than RS3 in rice. More importantly, *Wx* regulates all subtypes of RS in gene interaction networks, which is not only further demonstrated the main effect of *Wx* on RS, but also revealed that some minor genes are also involved in the regulatory effect of major genes on RS synthesis.

The genes regulating starch biosynthesis in rice endosperm were differentially expressed at different developmental stages of fresh seeds. Most starch biosynthesis-related proteins were increased at 6 - 20 DAF and decreased upon the high-temperature conditions (Tappiban et al., 2021). Besides that, grain quality influenced the dynamic expression of SSRGs, which were most expressed at lower level in inferior grains at early filling stage, while at late filling stage, the expression of those genes was higher in inferior grains and lower in superior grains (Sun et al., 2015). This study found that the gene expression levels of *Wx*, *SSI*, *SSIIa* and *PUL* gradually increased from 7 to 17 DAF, and decreased at 22 DAF. The *Wx* gene and the two subunits of *AGPase* had higher expression levels at both 10 and 14 DAF than that at 6 DAF, and with the highest mRNA levels at 14 DAF for the high AAC cultivars (Biselli et al., 2015). Therefore, the expression levels of related genes increased at early filling stage and then decreased at late filling stage for the varieties with high AAC and high RS. Expression levels of *Wx*, *SSI*, and *SSIIa* at 17 DAF were significantly positively correlated with both of RSm and RSa, and it showed that the high expression of the regulated genes at early filling stage contributed to the formation of RS. However, few transcripts of *SSIIb* were detected in the seeds. Previous study showed that the highest expression level of *SSIIb* was in the mature leaves, but its expression was lower in the developing seeds (Li et al., 2018). Even so, *SSIIb* still had distinct functions during the starch biosynthesis and it have been proved that introduction of the *SSIIb RNAi* cassette decreased AAC and intermediate chains with DP 13–24, but marked increased short amylopectin chains with DP 6–12, resulting in decreased GT (Lu et al., 2023). Furthermore, the improved grain quality of *SSIIb RNAi* transgenic lines was achieved by coordinated downregulating the expression of *SSIIb*, *SSIIa* and *Wx* (Huang et al., 2021). The genetic regulatory effect of *SSIIb* on RS might result from the transient starch produced in the leaves. In addition, the results of expression analysis for the *Wx*, *SSI*, and *SSIIa* and *PUL* indicated that the translation of these genes were subjected to the same signal instruction at the developmental stage, and interacting with each other.

In principle, starch granules were constructed by amorphous and crystalline layers alternately, which were composed of amylose and amylopectin, and they were mostly polygons with small size, irregular shape and obvious edges. Previous researches indicated that certain starch granules with special forms existed in high-RS varieties. The starch granules of rice mutants with high - RSc content, such as *RS111*, *B10*, and *MR4* were predominantly spherical or oval (Yang et al., 2005; Shu et al., 2014; Zhou et al., 2016), whereas the starch granules of R7954 and Zhong9B with high - RSm content were pentagonal and angular (Yang et al., 2005). Starch granules of mutants with high AC, *be2b* and *be1be2b*, were larger irregularly shaped compared with WT cultivars Taichung 65 and *be1* mutant (Miura et al., 2021). Besides, the irregular rod-shaped starch granules existed in lines with high - AC such as rice *be1be2b* and *ss3a be2b* (Asai et al., 2014). The starch granules in the *SSIIIa SSVb* double mutant are small and spherical compared with those in the wild type and *SSIIIa* (Toyosawa et al., 2016). The starch granules of eight rice germplasm with different RS were predominantly polyhedral and angular shape (**Figure 5**), similar to the structure of R7954 and Zhong9B granules. Moreover, RSc, RSr and RSb were significantly positively correlated with medium-sized starch granules for the most cultivars. As a result, RSc and RSr were more likely to be contained in medium and small starch granules.

**Figure 7:**
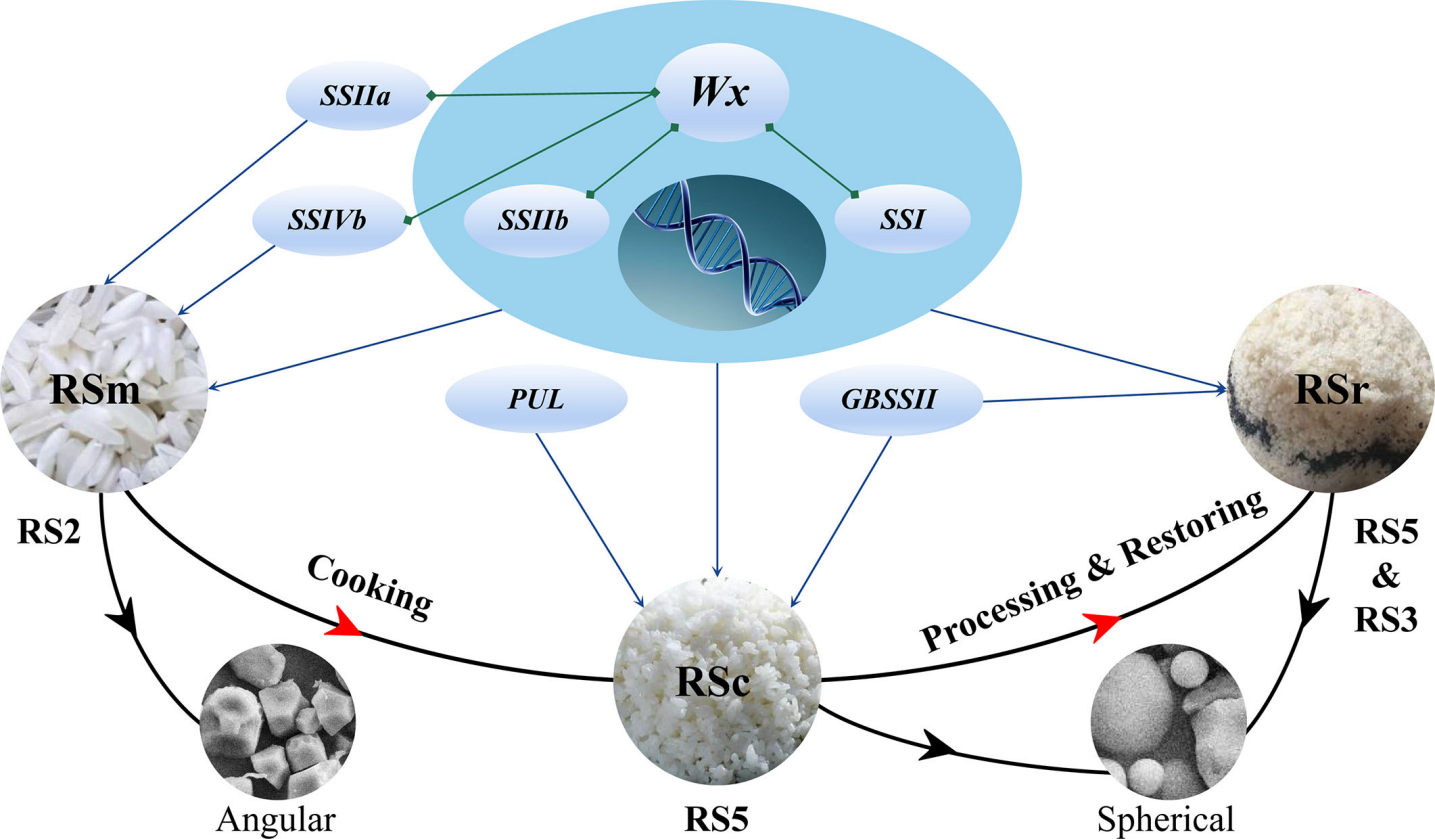
The regulating mechanism of RS in various processing statuses. RSm is the resistant starch in raw milled rice; RSc is the resistant starch in cooked rice; and RSr is the resistant starch in retrograded rice.

Overall, RSm, RSc and RSr had significant variation and different genetic regulation mechanisms by measuring them in same sample and analyzing the population structure and genetic relationship for the rice accessions with moderate or high AAC (**Figure 7**). *Wx* was the key factor for the formation of RS, especially the RSc and RSr. RSm, RSc, and RSr were co-regulated by *Wx*, *SSIIb* and *SSI*. In addition, RSm was also regulated by *SSIIa* and *SSIVb*, while *GBSSII* was associated with the genetic regulation of RSc and RSr, and *PUL* only acted on RSr. Besides that, as the center of genetic regulation on RS, *Wx* also significantly interacted with *SSIIa*, *SSI*, *SSIIb* and *SSIVb*. There was different interaction mechanisms for RSm, RSc and RSr, and RS2 was more complex than RS3. The high expression of *Wx* and its significantly positive correlation with RSm, RSa, and RSb indicated its important effect during the RS biosynthesis. The highest expression of *Wx*, *SSI*, and *SSIIa* at 17 DAF were beneficial to formation of RS. The highly positive correlation of expression levels among genes implied their interactions with each other. In addition to AAC and GT being solid predictors of RS, TPr was firstly found to have a significant negative correlation with RS under different processing statuses. Starch granules with high RSm should have a higher ratio of polyhedral and angular shape. RSc and RSr were more likely to be contained in starch granules of medium and small size. ‘Guichao2’, ‘STLT’, ‘CG133R’ and ‘Shuhui316’ contained high RSc or RSr, which would retain more RS after cooking and could be selected as core resources for the molecular breeding with high - RS. The above discovery is of great significance to understand the genetic mechanism for the biosynthesis of different subtype RS and further explore the reason for variation of starch granules characteristics.

## Data availability statement

The datasets presented in this study can be found in online repositories. The names of the repository/repositories and accession number(s) can be found in the article/**Supplementary Material**.

## Author contributions

CL: Conceptualization, Data curation, Investigation, Writing – original draft, Writing – review & editing. HX: Methodology, Software, Writing – review & editing. HY: Formal analysis, Investigation, Methodology, Writing – review & editing. OZ: Data curation, Formal analysis, Software, Writing – review & editing. YHa: Data curation, Formal analysis, Writing – review & editing. QL: Investigation, Supervision, Validation, Writing – review & editing. YHu: Funding acquisition, Project administration, Supervision, Resources, Writing – review & editing. XX: Conceptualization, Funding acquisition, Investigation, Project administration, Resources, Supervision, Validation, Writing – review & editing.
