# Supplementary material for: Physicochemical properties and molecular mechanisms of different resistant starch subtypes in rice
Source: Front Plant Sci. 2024 Jan 8;14:1313640. doi: 10.3389/fpls.2023.1313640 (PMC10800921; doi:10.3389/fpls.2023.1313640)
Supplement: Supplementary file 1 [file DataSheet_1.docx]

Supplementary data

[Supplementary figure1 Population structure of 73 rice accessions.](#OLE_LINK1)

[Supplementary figure2 Cluster analysis based on genetic similarity coefficient.](#OLE_LINK2)

[Supplementary figure3 Heat map of kinship.](#OLE_LINK3)

[Supplementary table1 The name of 73 rice materials.](#OLE_LINK5)

[Supplementary table2 Information of 42 pairs of SSR markers.](#OLE_LINK6)

[Supplementary table3 Primer information of 18 starch synthesis-related genes.](#OLE_LINK7)

[Supplementary table4 Primer information of Real-time PCR.](#OLE_LINK8)

[Supplementary table5 The genetic diversity of SSR markers in rice.](#OLE_LINK11)

[Supplementary table6 The Q value of 73 rice materials.](#OLE_LINK12)

[Supplementary table7 The results of gene genotyping for 73 rice accessions.](#OLE_LINK13)

| 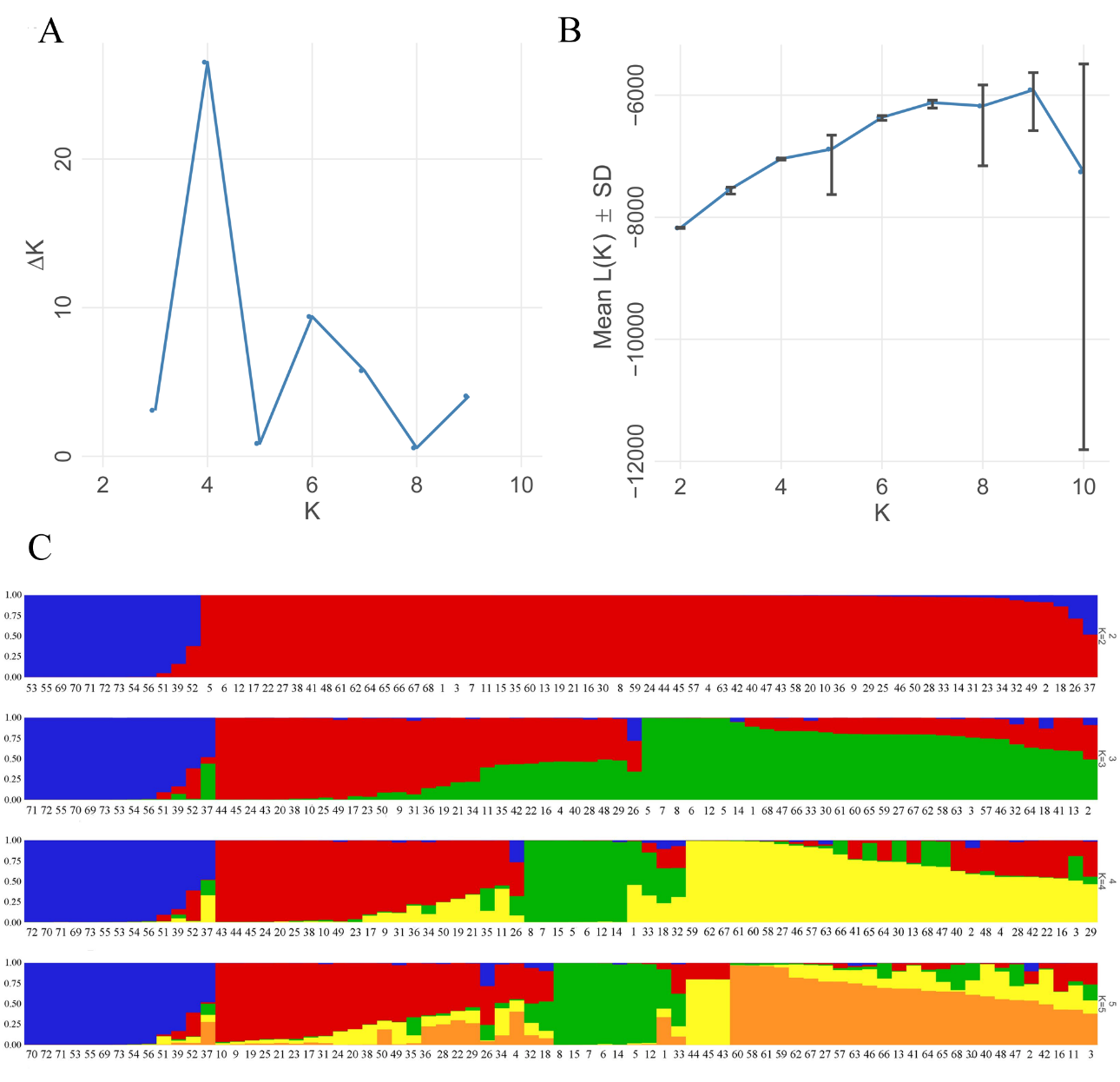 |
| --- |
| **Fig. S1.** Population structure of 73 rice accessions. (A) Line chart of Mean L (K) with the change of K; (B) Line chart of ΔK with the change of K; (C) Population structure of 73 rice accessions. Blue, red, green and yellow represents four subgroups, respectively. The numbers are sample symbols corresponding to Table S1. |

| 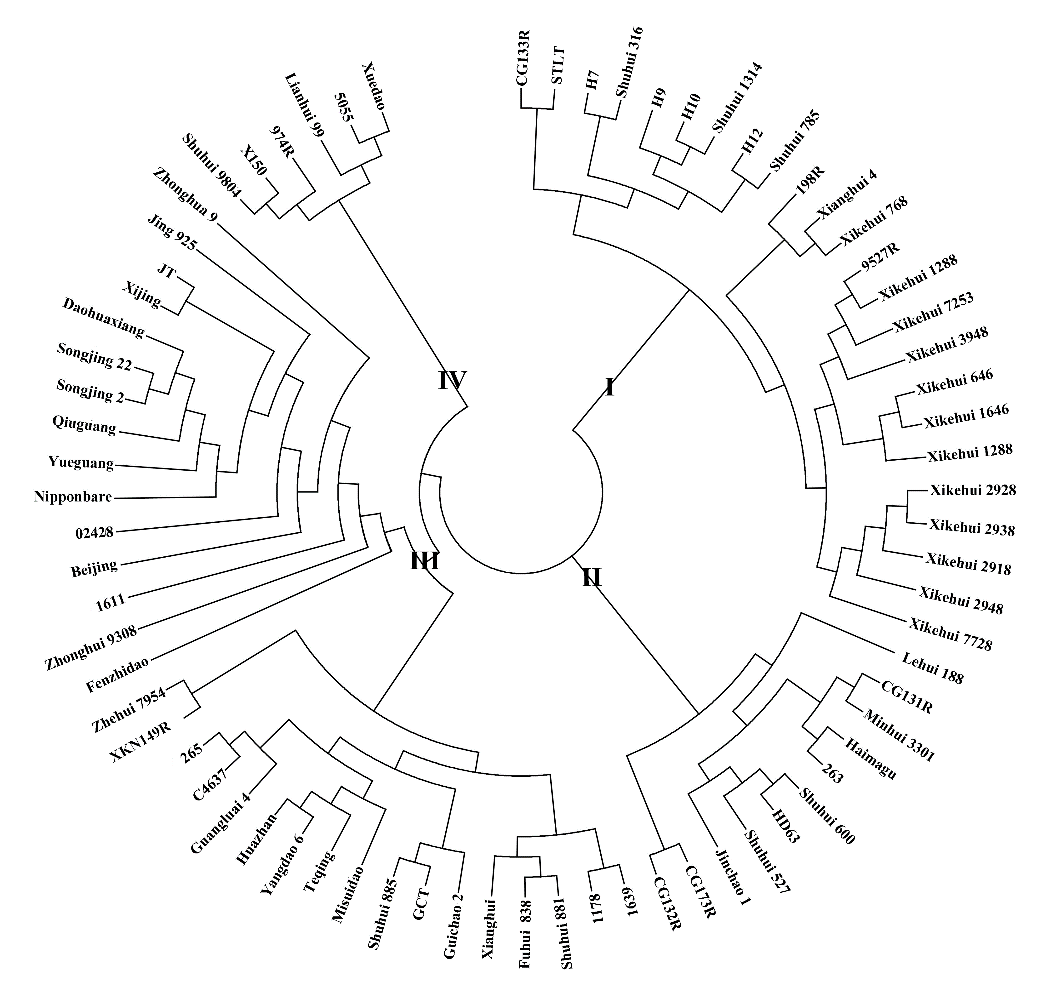 |
| --- |
| **Fig. S2.** Cluster analysis based on genetic similarity coefficient. |

| 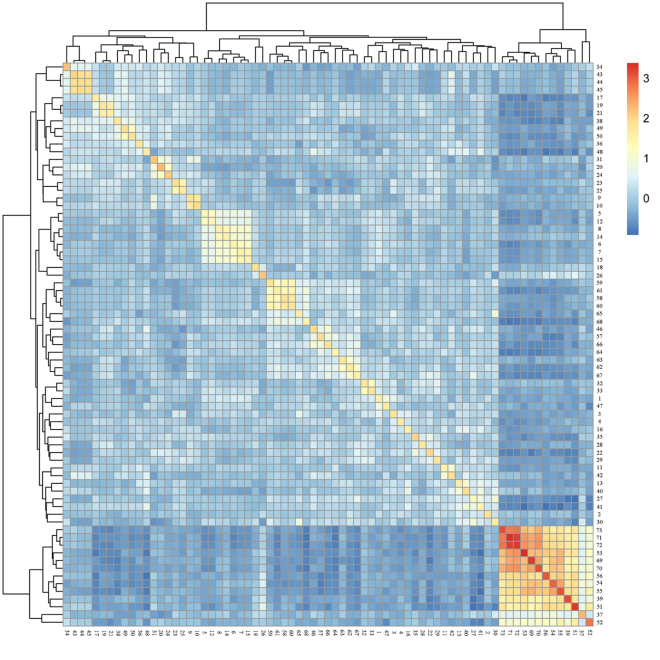 |
| --- |
| **Fig. S3.** Heat map of kinship. The numbers are sample symbols corresponding to Table S1. |

| **Table S1**  The name of 73 rice materials. |
| --- |
| \| No. \| Lines \| No. \| Lines \| No. \| Lines \| No. \| Lines \| \| --- \| --- \| --- \| --- \| --- \| --- \| --- \| --- \| \| 1 \| CG133R \| 20 \| Shuhui 881 \| 39 \| Beijing \| 58 \| Xikehui 2928 \| \| 2 \| Lehui188 \| 21 \| Shuhui 885 \| 40 \| HD63 \| 59 \| Xikehui 2918 \| \| 3 \| CG173R \| 22 \| Shuhui 9804 \| 41 \| Shuhui 527 \| 60 \| Xikehui 2938 \| \| 4 \| CG132R \| 23 \| XKN149R \| 42 \| Minhui 3301 \| 61 \| Xikehui 2948 \| \| 5 \| H7 \| 24 \| Fuhui 838 \| 43 \| Teqing \| 62 \| Xikehui 646 \| \| 6 \| H9 \| 25 \| Zhehui 7954 \| 44 \| Yangdao 6 \| 63 \| Xikehui 1646 \| \| 7 \| H10 \| 26 \| Zhonghui 9308 \| 45 \| Huazhan \| 64 \| Xikehui 3948 \| \| 8 \| H12 \| 27 \| 198R \| 46 \| 9527R \| 65 \| Xikehui 768 \| \| 9 \| 1639 \| 28 \| 974R \| 47 \| STLT \| 66 \| Xikehui 1288 \| \| 10 \| 1178 \| 29 \| Lianhui99 \| 48 \| 263 \| 67 \| Xikehui 1288 \| \| 11 \| CG131R \| 30 \| Xianghui 4 \| 49 \| C4637 \| 68 \| Xikehui 7728 \| \| 12 \| Shuhui 316 \| 31 \| Xianghui \| 50 \| 265 \| 69 \| Yueguang \| \| 13 \| Shuhui 600 \| 32 \| 5055 \| 51 \| 02428 \| 70 \| Qiuguang \| \| 14 \| Shuhui 785 \| 33 \| Xuedao \| 52 \| Zhonghua 9 \| 71 \| Songjing 2 \| \| 15 \| Shuhui 1314 \| 34 \| Misuidao \| 53 \| Nipponbare \| 72 \| Songjing 22 \| \| 16 \| Jinchao 1 \| 35 \| X150 \| 54 \| Xijing \| 73 \| Daohuaxiang \| \| 17 \| Guichao 2 \| 36 \| Haimagu \| 55 \| JT \|  \|  \| \| 18 \| Fenzhidao \| 37 \| 1611 \| 56 \| Jing 925 \|  \|  \| \| 19 \| GCT \| 38 \| Guangluai 4 \| 57 \| Xikehui 7253 \|  \|  \| |

| **Table S2**  Information of 42 pairs of SSR markers. |
| --- |
| \| Chr. \| Primers \| Chr. \| Primers \| \| --- \| --- \| --- \| --- \| \| 1 \| RM5, RM128, RM259 \| 7 \| RM11, RM180, RM234, RM336 \| \| 2 \| RM211, RM263, RM475, RM525 \| 8 \| RM223, RM264, RM308 \| \| 3 \| RM251, RM489, RM514, RM520 \| 9 \| RM205, RM242, RM316, RM566, OSR28 \| \| 4 \| RM252, RM255, RM335, RM471 \| 10 \| RM216, RM258, RM333, RM474 \| \| 5 \| RM122, RM289 \| 11 \| RM332, RM286 \| \| 6 \| RM412, RM528, RM585, RM587 \| 12 \| RM101, RM17, RM519 \| |

| **Table S3**  Primer information of 18 starch synthesis-related genes.   \| Gene \| Molecular marker \| \| Sequence of primers (5′→3′) \| Marker type \| \| --- \| --- \| --- \| --- \| --- \| \| *AGPlar* \| *AGPlar* M1 \| \| [F] CGTTCAGGTTCAGGCAATCA  [R] GGAAGGGTGGTGATGTGGAG \| STS \| \| *AGPlar* M2 \| \| [F] GCGTGAACTGAACATCCATCT  [R] GGTTCAAGCCTTCAGGTCAG \| CAPS (*Tsp*4*5*I ) \| \| *AGPiso* \| *AGPiso* M2 \| \| [F] CAATCGCTGCCATCGGTTG  [R] TTCCACATCGTTAGGTACACG \| STS \| \| *AGPiso* M3 \| \| [F] TGGAATGGGAACTCTATTATTGG  [R] TCCCAACCTCTACCTTCAAATG \| CAPS (*Eco* RI) \| \| *AGPsma* \| *AGPsma* M1 \| \| [F] TCTATTCTCAGCCCTCCAACC  [R] GTGTGTTTAGAGGTGCTTTTCG \| STS \| \| *AGPsma* M2 \| \| [F] GTGTGTTTAGAGGTGCTTTTCG  [R] TATCTTCCCAGTAACCATCA \| STS \| \| *GBSSII* \| *GBSSII* \| \| [F] TTGCTGCGAATTATCTGCG  [R] ACCTCCTCCCACTTCTTTGC \| STS \| \| *SSI* \| *SSI* M1 \| \| [F] GGTAGGGTAGGTCAATCTGGC  [R] ATAGAGAAGACAATGTGGCAACC \| CAPS (*Nru* I) \| \| *SSI* M2 \| \| [F] CTTCTATCCATTCCTTAATCCCA  [R] ATGCTATTGATGTTAAGAGGGC \| STS \| \| *SSI* M3 \| \| [F] GACCCACCTCGCTATCTGTTG  [R] GGAAACACCAGACATCAACCAG \| CAPS (*Apa* I ) \| \| *SSII-1* \| *SSII-1* M1 \| \| [F] CACCCCACCGTTCTACTATGC  [R] TCCATAGTTTCATTGAGATTGCTC \| STS \| \| *SSII-1* M2 \| \| [F] CAAGTTGGTGACGATAGTGATGA  [R] AACAGAGCCTCCATTACCTTTAC \| CAPS (*Age* I) \| \|  \| *SSII-1* M3 \| \| [F] AGAGATCAAATCGTGGAAC  [R] TGGAGTGAAGTAGTGGAAT \| STS \| \|  \| *SSII-1* M4 \| [F] ATCTTTAGACGATTAGCG  [R] AAGTCACAAGTAGAAGGG \| \| STS \| \| \| *SSII-2* \| *SSII-2* M1 \| [F] AGATTTGAACTCAGGACTTGGTG  [R] TCTATGGGCTCTATCCTTACTAGG \| \| STS \| \| \| *SSII-2* M2 \| [F] CGCTCGTTGCCTAGCTAGC  [R] GGCGAGGAAGCGATTGCC \| \| STS \| \| \| *SSII-2* M3 \| [F] ACAGTATGTTTGCCTCAGCG  [R] GTAAATCCACCCAGCCAGTC \| \| STS \| \| \| *SSII-3* \| *SSII-3* M1 \| [F]CCAATACCGTAAACTAGCGACTATG  [R] TACAGGTAGAATGGCAGTGGTG \| \| STS \| \| \| *SSII-3* M2 \| [F] GGTTCTCGGTGAAGATGGC  [R] GTGGTCCCAGCTGAGGTCC \| \| CAPS (*Ban* II) \| \| \| *SSII-3* M3 \| [F] AACTGACTCATACACGGATAACG  [R] CACGCACGAACGGAAACC \| \| CAPS (*Nhe* I) \| \| \| *SSIII-1* \| *SSIII-1* M1 \| [F] AAGAAGGGAAGGGAGTCAGC  [R] GCCATCTCCATTGCCAGC \| \| STS \| \| \| *SSIII-1* M2 \| [F] CAAGCAATGATTCAGGCACA  [R] GGAGACAGGAGCAAAAGGC \| \| CAPS (*Eco* RI) \| \| \| *SSIII-1* M3 \| [F] CAAATCAACTGTAAGTGCTGGAG  [R] GAGAACGGAGAAAATGGCAT \| \| CAPS *(Nde* I) \| \| \| *SSIII-1* t1 \| [F] GGAGCAATAGGTGGTTCAA  [R] GCCAAATCTACTCTCGTCA \| \| CAPS (*Eco* R72I) \| \| \| *SSIII-2* \| *SSIII-2 CAPS* \| [F] TTGGAACTTTGGTTGGTATATCG  [R] CTTACCTTTGCAATGGGTGC \| \| CAPS (*Mlu* C I) \| \| \| *SSIV-1* \| *SSIV-1* M1 \| [F] CATTGTGTCTTGAAGTCTGTGCT  [R] CGATGGGTTAGTGCTGTGG \| \| CAPS (*Nde* I) \| \| \| *SSIV-2* \| *SSIV-2* M1 \| [F] CTTCTGATTGATGGTTGGTTGC  [R]GGAAGAATAATCTCTACTAGGTGGC \| \| CAPS (*Sph*I) \| \| \| *SSIV-2* M2 \| [F] TTCCCTTGGTGGTGCGTG  [R] TAAAGCGTTCCGACAGTA \| \| STS \| \| \| *SSIV-2* M3 \| [F] TCAAGTATGGTTTACCTATG  [R] TTTCCCAATGACTTCTAA \| \| CAPS (*Eco*72I) \| \| \| *SBE1* \| *SBE1* M1 \| [F] GCTACATAACACGCATACAAAGT  [R]AGACAAAAGCGAAAGGTAATGAG \| \| STS \| \| \| *SBE1* M2 \| [F] GTGGGGAAAACAAGTAAGTCTG  [R] AGTTCCATCAGAAGAATCAGGG \| \| STS \| \| \| *SBE1* M3 \| [F] GGAAATGGGAGTCGCC  [R] CGAAGAAACCACGCTCA \| \| STS \| \| \| *SBE1* M4 \| [F] ATTGTTGCTGAAGATGTTT  [R] ACGGTTGATGGTAGGTG \| \| CAPS (*Taq* I) \| \| \| *SBE3* \| *SBE3* M1 \| [F] AAGGTTAGCATTGGTTGGTGAG  [R] TCTCCTTGAACAGCGACAGC \| \| STS \| \| \| *SBE3* t1 \| [F] TTCCATTATTTCTTTGCA  [R] TATCCTCCCTGAACCAC \| \| STS \| \| \| *SBE4* \| *SBE4*M1 \| [F] CACCAATTATATTAGCGTGCTCC  [R] CGTGGCTCTTGGCTCTCTTG \| \| STS \| \| \| *SBE4* M2 \| [F] CCATCACCTCAAATACATCACTC  [R] AGACTGGAATGCCCCTTAGG \| \| STS \| \| \| *ISA* \| *ISA* M1 \| [F] ATAGATGCTAATGTGATGTGGC  [R] TGGTATAGGCACAACCGTAGA \| \| STS \| \| \| *ISA* M2 \| [F] ACAAGCACACGACACCTA  [R] CAACAAACCAAACTCATT \| \| CAPS (*Hind* III) \| \| \| *ISA* M3 \| [F] TGTGGGAATACCTTCAACTG  [R] ATAAAACCCTTACAGGCTTG \| \| STS \| \| \| *PUL* \| *PUL* M2 \| [F] GACAACCGTCCGCTTTAGTTTC  [R] GCATTTGAGAGGGTTTGGATTC \| \| STS \| \| \| *PUL* M6 \| [F] ATTTAACTGTATGGACTGAG  [R] GATACCAACCAAACAAGA \| \| STS \| \| \| *Wx* \| Wx M1  PCR-ACCI  Wx EX10 \| [F] CACAGCAACAGCTAGACAACCAC  [R] CACGACGACGGAGGGGAAC  [F] GCTTCACTTCTCTGCTTGTG  [R] ATGATTTAACGAGAGTTAA  [F] GCATCACCGGCATCGTC  [R] GCTCCGGCCATGATGAGATG \| \| STS  CAPS (*ACC*I)  CAPS (*Apa*I) \| \| |
| --- | --- | --- | --- | --- | --- | --- | --- | --- | --- | --- | --- | --- | --- | --- | --- | --- | --- | --- | --- | --- | --- | --- | --- | --- | --- | --- | --- | --- | --- | --- | --- | --- | --- | --- | --- | --- | --- | --- | --- | --- | --- | --- | --- | --- | --- | --- | --- | --- | --- | --- | --- | --- | --- | --- | --- | --- | --- | --- | --- | --- | --- | --- | --- | --- | --- | --- | --- | --- | --- | --- | --- | --- | --- | --- | --- | --- | --- | --- | --- | --- | --- | --- | --- | --- | --- | --- | --- | --- | --- | --- | --- | --- | --- | --- | --- | --- | --- | --- | --- | --- | --- | --- | --- | --- | --- | --- | --- | --- | --- | --- | --- | --- | --- | --- | --- | --- | --- | --- | --- | --- | --- | --- | --- | --- | --- | --- | --- | --- | --- | --- | --- | --- | --- | --- | --- | --- | --- | --- | --- | --- | --- | --- | --- | --- | --- | --- | --- | --- | --- | --- | --- | --- | --- | --- | --- | --- | --- | --- | --- | --- | --- | --- | --- | --- | --- | --- | --- | --- | --- | --- | --- | --- | --- | --- | --- | --- | --- | --- | --- | --- | --- | --- | --- | --- | --- | --- | --- | --- | --- | --- | --- | --- | --- | --- | --- | --- | --- | --- | --- | --- | --- | --- | --- | --- | --- | --- | --- | --- | --- | --- | --- | --- | --- | --- | --- | --- | --- | --- | --- | --- | --- | --- | --- | --- | --- | --- | --- |

| **Table S4**  Primer information of Real-time PCR. |
| --- |
| \| Gene \| Type \| Primer \| Locus ID \| \| --- \| --- \| --- \| --- \| \| *Actin* \| Real-time PCR \| F:TCAGCAACTGGGATGATATGGAG  R:GCCGTTGTGGTGAATGAGTAAC \| LOC4333919 \| \| *Wx* \| Real-time PCR \| [F] 5'-TTCCTGGAGAAGGTTTGGG-3'  [R] 5'-AACGCATCTGGTTGTCTTTG-3' \| LOC4340018 \| \| *SSI* \| Real-time PCR \| [F] 5’ CCAGTCTTGTGCCAGTCCTT 3’  [R] 5’ ATTCAGGTGGCAATCCCAGG 3’ \| LOC9269493 \| \| *SSII-2* \| Real-time PCR \| [F] 5’ ACAGGGTAGCTGGACAGGAT 3’  [R] 5’ CATTGTGCCGGTGTCGAAAG 3’ \| LOC4330709 \| \| *SSII-3* \| Real-time PCR \| [F] 5’ GGCTGCTGAATGTTCTCCCT 3’  [R] 5’ CCGCGTAATCACCGTACCTT 3’ \| LOC4340567 \| \| *PUL* \| Real-time PCR \| [F] 5’ GTGGCTGAGAAGTTCCCGTT 3’  [R] 5’TCTTGATGCCTCCCATGAGC 3’ \| LOC4335042 \| |

| **Table S5**  The genetic diversity of SSR markers in rice. |
| --- |
| \| Marker \| Allele numbers \| PIC \| Marker \| Allele numbers \| PIC \| Marker \| Allele numbers \| PIC \| \| --- \| --- \| --- \| --- \| --- \| --- \| --- \| --- \| --- \| \| RM5 \| 3 \| 0.465 \| RM122 \| 3 \| 0.488 \| RM316 \| 2 \| 0.223 \| \| RM128 \| 3 \| 0.456 \| RM289 \| 3 \| 0.530 \| RM566 \| 4 \| 0.533 \| \| RM259 \| 3 \| 0.511 \| RM412 \| 3 \| 0.223 \| OSR28 \| 5 \| 0.665 \| \| RM211 \| 3 \| 0.375 \| RM528 \| 4 \| 0.592 \| RM216 \| 2 \| 0.273 \| \| RM263 \| 5 \| 0.585 \| RM585 \| 6 \| 0.603 \| RM258 \| 3 \| 0.478 \| \| RM475 \| 4 \| 0.676 \| RM587 \| 3 \| 0.490 \| RM333 \| 8 \| 0.692 \| \| RM525 \| 6 \| 0.499 \| RM11 \| 4 \| 0.594 \| RM474 \| 7 \| 0.647 \| \| RM251 \| 5 \| 0.595 \| RM180 \| 2 \| 0.209 \| RM332 \| 4 \| 0.480 \| \| RM489 \| 2 \| 0.273 \| RM234 \| 2 \| 0.262 \| RM286 \| 3 \| 0.581 \| \| RM514 \| 6 \| 0.703 \| RM336 \| 5 \| 0.503 \| RM101 \| 3 \| 0.285 \| \| RM520 \| 3 \| 0.507 \| RM223 \| 4 \| 0.406 \| RM17 \| 3 \| 0.393 \| \| RM252 \| 4 \| 0.588 \| RM264 \| 4 \| 0.604 \| RM519 \| 2 \| 0.268 \| \| RM255 \| 3 \| 0.554 \| RM308 \| 2 \| 0.027 \| Mean \| 3.786 \| 0.474 \| \| RM335 \| 5 \| 0.751 \| RM205 \| 5 \| 0.416 \|  \|  \|  \| \| RM471 \| 2 \| 0.176 \| RM242 \| 6 \| 0.723 \|  \|  \|  \|   PIC, polymorphism information content. |

| **Table S6**  The Q value of 73 rice accessions. |
| --- |
| \| No. \| Cluster1 \| Cluster2 \| Cluster3 \| Cluster4 \| No. \| Cluster1 \| Cluster2 \| Cluster3 \| Cluster4 \| \| --- \| --- \| --- \| --- \| --- \| --- \| --- \| --- \| --- \| --- \| \| 1 \| 0.002 \| 0.008 \| 0.534 \| 0.456 \| 38 \| 0.001 \| 0.976 \| 0.010 \| 0.013 \| \| 2 \| 0.095 \| 0.307 \| 0.010 \| 0.589 \| 39 \| 0.838 \| 0.068 \| 0.044 \| 0.049 \| \| 3 \| 0.002 \| 0.191 \| 0.296 \| 0.511 \| 40 \| 0.007 \| 0.364 \| 0.003 \| 0.626 \| \| 4 \| 0.005 \| 0.426 \| 0.016 \| 0.553 \| 41 \| 0.001 \| 0.227 \| 0.009 \| 0.763 \| \| 5 \| 0.001 \| 0.003 \| 0.994 \| 0.003 \| 42 \| 0.007 \| 0.434 \| 0.007 \| 0.553 \| \| 6 \| 0.001 \| 0.005 \| 0.991 \| 0.004 \| 43 \| 0.002 \| 0.992 \| 0.003 \| 0.002 \| \| 7 \| 0.001 \| 0.002 \| 0.996 \| 0.002 \| 44 \| 0.002 \| 0.992 \| 0.004 \| 0.002 \| \| 8 \| 0.001 \| 0.002 \| 0.996 \| 0.002 \| 45 \| 0.002 \| 0.992 \| 0.003 \| 0.002 \| \| 9 \| 0.007 \| 0.861 \| 0.013 \| 0.118 \| 46 \| 0.02 \| 0.024 \| 0.020 \| 0.936 \| \| 10 \| 0.003 \| 0.967 \| 0.019 \| 0.011 \| 47 \| 0.006 \| 0.01 \| 0.308 \| 0.676 \| \| 11 \| 0.002 \| 0.549 \| 0.04 \| 0.409 \| 48 \| 0.001 \| 0.373 \| 0.049 \| 0.576 \| \| 12 \| 0.001 \| 0.004 \| 0.986 \| 0.009 \| 49 \| 0.02 \| 0.967 \| 0.005 \| 0.008 \| \| 13 \| 0.003 \| 0.273 \| 0.016 \| 0.707 \| 50 \| 0.003 \| 0.748 \| 0.003 \| 0.245 \| \| 14 \| 0.022 \| 0.002 \| 0.973 \| 0.003 \| 51 \| 0.907 \| 0.078 \| 0.004 \| 0.011 \| \| 15 \| 0.001 \| 0.002 \| 0.995 \| 0.002 \| 52 \| 0.611 \| 0.370 \| 0.005 \| 0.014 \| \| 16 \| 0.002 \| 0.458 \| 0.005 \| 0.535 \| 53 \| 0.996 \| 0.001 \| 0.002 \| 0.001 \| \| 17 \| 0.001 \| 0.903 \| 0.015 \| 0.081 \| 54 \| 0.990 \| 0.002 \| 0.005 \| 0.002 \| \| 18 \| 0.106 \| 0.235 \| 0.426 \| 0.233 \| 55 \| 0.996 \| 0.001 \| 0.002 \| 0.001 \| \| 19 \| 0.002 \| 0.709 \| 0.005 \| 0.285 \| 56 \| 0.984 \| 0.003 \| 0.008 \| 0.006 \| \| 20 \| 0.003 \| 0.985 \| 0.002 \| 0.010 \| 57 \| 0.006 \| 0.064 \| 0.013 \| 0.917 \| \| 21 \| 0.002 \| 0.655 \| 0.005 \| 0.338 \| 58 \| 0.007 \| 0.005 \| 0.007 \| 0.981 \| \| 22 \| 0.002 \| 0.448 \| 0.007 \| 0.544 \| 59 \| 0.002 \| 0.002 \| 0.003 \| 0.994 \| \| 23 \| 0.004 \| 0.958 \| 0.034 \| 0.004 \| 60 \| 0.002 \| 0.003 \| 0.008 \| 0.987 \| \| 24 \| 0.001 \| 0.992 \| 0.005 \| 0.002 \| 61 \| 0.001 \| 0.002 \| 0.007 \| 0.990 \| \| 25 \| 0.002 \| 0.979 \| 0.012 \| 0.006 \| 62 \| 0.001 \| 0.004 \| 0.003 \| 0.992 \| \| 26 \| 0.268 \| 0.412 \| 0.235 \| 0.085 \| 63 \| 0.049 \| 0.017 \| 0.032 \| 0.901 \| \| 27 \| 0.001 \| 0.014 \| 0.030 \| 0.955 \| 64 \| 0.001 \| 0.245 \| 0.014 \| 0.739 \| \| 28 \| 0.023 \| 0.411 \| 0.014 \| 0.553 \| 65 \| 0.002 \| 0.032 \| 0.215 \| 0.752 \| \| 29 \| 0.015 \| 0.429 \| 0.090 \| 0.465 \| 66 \| 0.001 \| 0.003 \| 0.169 \| 0.827 \| \| 30 \| 0.002 \| 0.003 \| 0.257 \| 0.738 \| 67 \| 0.001 \| 0.004 \| 0.003 \| 0.992 \| \| 31 \| 0.027 \| 0.851 \| 0.008 \| 0.113 \| 68 \| 0.001 \| 0.007 \| 0.307 \| 0.685 \| \| 32 \| 0.070 \| 0.268 \| 0.354 \| 0.308 \| 69 \| 0.996 \| 0.001 \| 0.001 \| 0.001 \| \| 33 \| 0.032 \| 0.117 \| 0.524 \| 0.327 \| 70 \| 0.997 \| 0.001 \| 0.001 \| 0.001 \| \| 34 \| 0.024 \| 0.768 \| 0.107 \| 0.101 \| 71 \| 0.996 \| 0.001 \| 0.001 \| 0.002 \| \| 35 \| 0.003 \| 0.581 \| 0.276 \| 0.140 \| 72 \| 0.997 \| 0.001 \| 0.001 \| 0.001 \| \| 36 \| 0.007 \| 0.772 \| 0.016 \| 0.205 \| 73 \| 0.996 \| 0.001 \| 0.001 \| 0.001 \| \| 37 \| 0.479 \| 0.007 \| 0.185 \| 0.329 \|  \|  \|  \|  \|  \| |

| **Accession** | ***AGPlarM1*** | ***AGPlarM2*** | ***AGPisoM2*** | ***AGPisoM3*** | ***AGPsma M2*** | ***AGPsma 1*** | ***GBSSⅡM1*** | ***SSI M1*** | ***SSI M2*** | ***SSI M3*** | ***SSIIc M1*** | ***SSIIc M2*** | ***SSIIc M3*** | ***SSIIc M4*** | ***SSIIb M1*** | ***SSIIb M2*** | ***SSIIb M3*** | ***SSIIa M1*** | ***SSIIa M2*** | ***SSIIa M3*** | ***SSIIIb M1*** | ***SSIIIb M2*** | ***SSIIIb M3*** | ***SSIIIb t1*** | ***SSIIIa M1*** | ***SSIIIa M2*** | ***SSIVa*** | ***SSIVb M1*** | ***SSIVb M2*** | ***SSIVb M3*** | ***SBE1 M1*** | ***SBE1 M2*** | ***SBE1 M3*** | ***SBE2 M4*** | ***SBEIIb M1*** | ***SBEIIb t1*** | ***SBEIIa M1*** | ***SBEIIa M2*** | ***ISA M1*** | ***ISA M2*** | ***ISA M3*** | ***PUL M1*** | ***PUL M2*** | ***PUL M3*** | ***PUL M4*** | ***PUL M5*** | ***PUL M6*** | ***PCR ACCI*** |
| --- | --- | --- | --- | --- | --- | --- | --- | --- | --- | --- | --- | --- | --- | --- | --- | --- | --- | --- | --- | --- | --- | --- | --- | --- | --- | --- | --- | --- | --- | --- | --- | --- | --- | --- | --- | --- | --- | --- | --- | --- | --- | --- | --- | --- | --- | --- | --- | --- |
| 1 | a | b | a | a | a | a | a | a | a | a | a | b | a | a | a | a | a | a | a | b | a | b | b | b | b | a | a | a | a | a | a | a | a | a | a | a | a | a | a | a | a | a | a | a | a | a | a | b |
| 2 | a | b | a | a | a | a | b | a | a | a | a | b | a | a | a | a | a | a | a | b | b | b | b | a | b | a | a | b | a | b | a | a | a | a | a | a | a | a | a | a | a | a | a | a | a | a | a | a |
| 3 | a | b | a | a | a | a | a | b | a | a | a | b | a | a | a | a | a | b | a | a | a | b | b | b | b | a | a | b | a | b | a | a | a | a | a | a | a | a | a | a | a | a | b | a | a | a | a | b |
| 4 | a | b | a | a | a | a | a | a | a | a | a | b | a | a | a | a | a | a | a | b | a | a | b | b | b | a | a | a | a | a | a | a | a | a | b | b | a | a | a | a | a | a | b | a | a | a | a | b |
| 5 | a | b | a | a | a | a | b | a | a | a | a | b | a | a | a | a | a | b | a | a | b | b | b | a | b | a | a | b | a | b | a | a | a | a | b | b | a | a | a | a | a | a | b | a | a | a | a | b |
| 6 | a | b | a | a | a | a | b | a | a | a | a | b | a | a | a | a | a | b | a | a | b | b | b | a | b | a | b | b | a | b | a | a | a | a | b | b | a | a | a | a | a | a | b | a | a | a | a | a |
| 7 | a | b | a | a | a | a | b | a | a | a | a | b | a | a | a | a | a | b | a | a | b | b | b | a | b | a | b | b | a | b | a | a | a | a | b | b | a | a | a | a | a | a | b | a | a | a | a | b |
| 8 | a | b | a | a | a | a | b | a | a | a | a | b | a | a | a | a | a | b | a | a | b | b | b | a | b | a | b | b | a | b | a | a | a | a | b | b | a | a | a | a | a | a | b | a | a | a | a | b |
| 9 | b | a | a | a | a | a | b | a | a | a | a | b | a | a | a | a | a | a | a | b | a | a | b | b | b | a | b | a | a | a | a | a | a | a | b | b | a | a | a | a | a | a | b | a | a | a | a | a |
| 10 | b | a | a | a | a | a | b | a | a | a | a | b | a | a | a | a | a | b | a | a | c | a | a | b | b | a | b | a | a | a | a | a | a | a | b | b | a | a | a | a | a | a | b | a | a | a | a | a |
| 11 | a | b | a | a | a | a | b | a | a | a | a | b | a | a | a | a | a | b | a | a | a | b | b | b | b | a | b | a | a | a | a | a | a | a | b | b | a | a | a | a | a | b | a | b | b | b | b | b |
| 12 | a | b | a | a | a | a | b | a | a | a | a | b | a | a | a | a | a | b | a | a | b | b | b | a | b | a | b | b | a | b | a | a | a | a | b | b | a | a | a | a | a | a | b | a | a | a | a | b |
| 13 | a | b | a | a | a | a | b | a | a | a | a | b | a | a | a | a | a | b | a | a | a | a | b | b | b | a | b | a | a | a | a | a | a | a | b | b | a | a | a | a | a | a | a | a | a | a | a | b |
| 14 | a | b | a | a | a | a | b | a | a | a | a | b | a | a | a | a | a | b | a | a | b | b | b | a | b | a | b | b | a | b | a | a | a | a | b | b | a | a | a | a | a | a | b | a | a | a | a | b |
| 15 | a | b | a | a | a | a | b | a | a | a | a | b | a | a | a | a | a | b | a | a | b | b | b | a | b | a | b | b | a | b | a | a | a | a | b | b | a | a | a | a | a | a | b | a | a | a | a | b |
| 16 | a | b | a | a | a | a | a | a | a | a | a | b | a | a | a | a | a | a | a | b | a | a | b | b | b | a | b | b | a | b | a | a | a | a | b | b | a | a | a | a | a | a | b | a | a | a | a | b |
| 17 | c | b | a | a | a | a | a | a | a | a | a | b | a | a | a | a | a | a | a | b | a | a | b | b | b | a | b | a | a | a | a | a | a | a | b | b | a | a | a | a | a | a | c | b | b | b | b | b |
| 18 | b | a | a | a | a | a | b | b | a | a | a | b | a | a | a | b | a | a | a | b | a | b | b | b | b | a | b | b | a | b | a | a | a | a | b | b | a | a | a | a | a | a | a | a | a | a | a | a |
| 19 | c | b | a | a | a | a | a | a | a | a | a | b | a | a | a | a | a | a | a | b | a | a | b | b | b | a | a | a | a | a | a | a | a | a | b | b | a | a | a | a | a | a | b | a | a | a | a | b |
| 20 | a | b | a | a | a | a | a | a | a | a | a | b | a | a | a | a | a | a | a | b | c | a | a | b | b | a | b | a | a | a | a | a | a | a | b | b | a | a | a | a | a | a | c | b | b | b | b | a |
| 21 | c | b | a | a | a | a | a | a | a | a | a | b | a | a | a | a | a | a | a | b | a | a | b | b | b | a | a | a | a | a | a | a | a | a | b | b | a | a | a | a | a | a | b | a | a | a | a | b |
| 22 | d | c | a | c | a | a | b | b | a | a | a | b | a | a | b | a | b | c | a | c | e | c | b | b | b | a | b | c | a | d | a | a | a | a | b | b | a | a | a | a | a | a | a | a | a | a | a | c |
| 23 | c | b | a | a | a | a | a | a | b | b | a | b | a | a | a | b | a | a | a | b | a | b | b | b | b | a | a | b | a | b | a | a | a | a | b | b | b | b | a | a | a | a | a | a | a | a | a | a |
| 24 | a | b | a | a | a | a | b | a | a | a | a | b | a | a | a | a | a | b | a | a | c | a | a | b | b | a | b | a | a | a | a | a | a | a | b | b | a | a | a | a | a | a | a | a | a | a | a | a |
| 25 | a | b | a | a | a | a | a | a | b | b | a | b | a | a | a | b | a | a | a | b | a | b | b | b | b | a | a | b | a | b | a | a | a | a | b | c | b | b | a | a | a | a | a | a | a | a | a | b |
| 26 | b | a | a | a | a | b | a | a | a | a | a | b | a | a | a | a | a | b | a | a | a | a | b | b | a | a | b | b | a | b | a | a | a | a | b | b | b | b | a | a | a | b | a | b | b | b | b | a |
| 27 | a | b | a | a | a | a | a | b | a | a | a | b | a | a | a | b | a | a | a | b | a | b | b | b | b | a | b | b | a | b | a | a | a | a | a | a | a | a | a | a | a | a | a | a | a | a | a | a |

**Table S7** The results of gene genotyping for 73 rice accessions.

**Table S7** Continued

| **Accession** | ***AGPlarM1*** | ***AGPlarM2*** | ***AGPisoM2*** | ***AGPisoM3*** | ***AGPsma M2*** | ***AGPsma 1*** | ***GBSSⅡM1*** | ***SSI M1*** | ***SSI M2*** | ***SSI M3*** | ***SSIIc M1*** | ***SSIIc M2*** | ***SSIIc M3*** | ***SSIIc M4*** | ***SSIIb M1*** | ***SSIIb M2*** | ***SSIIb M3*** | ***SSIIa M1*** | ***SSIIa M2*** | ***SSIIa M3*** | ***SSIIIb M1*** | ***SSIIIb M2*** | ***SSIIIb M3*** | ***SSIIIb t1*** | ***SSIIIa M1*** | ***SSIIIa M2*** | ***SSIVa*** | ***SSIVb M1*** | ***SSIVb M2*** | ***SSIVb M3*** | ***SBE1 M1*** | ***SBE1 M2*** | ***SBE1 M3*** | ***SBE2 M4*** | ***SBEIIb M1*** | ***SBEIIb t1*** | ***SBEIIa M1*** | ***SBEIIa M2*** | ***ISA M1*** | ***ISA M2*** | ***ISA M3*** | ***PUL M1*** | ***PUL M2*** | ***PUL M3*** | ***PUL M4*** | ***PUL M5*** | ***PUL M6*** | ***PCR ACCI*** |
| --- | --- | --- | --- | --- | --- | --- | --- | --- | --- | --- | --- | --- | --- | --- | --- | --- | --- | --- | --- | --- | --- | --- | --- | --- | --- | --- | --- | --- | --- | --- | --- | --- | --- | --- | --- | --- | --- | --- | --- | --- | --- | --- | --- | --- | --- | --- | --- | --- |
| 28 | b | a | a | a | a | a | b | a | a | a | a | b | a | a | a | a | a | b | a | a | c | a | a | b | b | a | b | b | a | b | a | a | a | a | b | b | a | a | a | a | a | a | b | a | a | a | a | a |
| 29 | d | c | a | a | a | a | b | a | a | a | a | b | a | a | c | a | c | b | a | a | e | c | b | b | b | a | b | c | a | d | a | a | a | a | a | a | a | a | a | a | a | a | a | a | a | a | a | a |
| 30 | a | b | a | a | a | a | b | b | a | a | a | b | a | a | a | b | a | a | a | b | a | b | b | b | b | a | b | b | a | b | a | a | a | a | a | a | a | a | a | a | a | a | a | a | a | a | a | a |
| 31 | d | c | a | a | a | a | b | b | a | a | a | b | a | a | a | a | a | b | a | a | c | a | a | b | b | a | b | a | a | a | a | a | a | a | b | b | a | a | a | a | a | a | a | a | a | a | a | a |
| 32 | a | b | a | a | a | a | b | b | a | a | a | b | a | a | a | a | a | a | a | b | a | b | b | b | b | a | b | b | a | b | a | c | a | c | a | a | a | a | a | a | a | c | d | a | a | a | a | a |
| 33 | a | b | a | a | a | a | b | a | a | a | a | b | a | a | a | a | a | a | a | b | a | b | b | b | b | a | b | b | a | b | a | c | a | c | a | a | a | a | a | a | a | c | d | a | a | a | a | a |
| 34 | a | b | a | c | a | a | b | a | a | a | a | b | a | a | a | a | a | a | a | b | e | c | c | b | b | a | b | b | a | b | a | a | a | a | b | b | b | b | a | a | a | a | c | b | b | b | b | a |
| 35 | d | c | b | b | a | a | b | b | a | a | a | b | a | a | c | a | c | c | a | c | a | a | b | b | b | a | b | a | a | a | a | a | a | a | b | b | a | a | a | a | a | a | a | a | a | a | a | c |
| 36 | b | a | a | a | a | a | b | a | a | a | a | b | a | a | a | a | a | a | a | b | a | a | b | b | b | a | b | a | a | a | a | a | a | a | b | b | a | a | a | a | a | a | b | a | a | a | a | b |
| 37 | a | b | a | a | a | b | b | b | a | a | a | b | a | a | a | b | a | a | a | b | a | b | b | b | b | a | b | b | a | b | a | a | a | a | a | a | b | b | a | a | a | a | a | a | a | a | a | a |
| 38 | c | b | a | a | a | a | b | a | b | b | a | a | b | b | a | a | a | b | a | a | c | b | a | b | b | a | b | a | a | a | a | a | a | a | a | a | a | a | a | a | a | a | c | b | b | b | b | b |
| 39 | b | a | a | a | b | b | a | b | a | a | b | a | a | a | a | a | a | a | a | b | b | b | b | b | b | b | a | b | a | b | a | a | a | b | a | a | b | b | b | b | b | a | a | b | b | b | b | b |
| 40 | b | a | a | a | a | a | b | a | b | b | a | b | a | a | a | a | a | b | a | a | a | a | b | b | b | a | b | a | a | a | a | a | a | a | b | b | a | a | a | a | a | a | b | a | a | a | a | a |
| 41 | a | b | a | a | a | a | b | a | a | a | a | b | a | a | a | a | a | b | a | a | a | a | b | b | b | a | b | a | a | a | a | a | a | a | a | a | a | a | a | a | a | a | a | a | a | a | a | b |
| 42 | a | b | a | a | a | a | b | a | a | a | a | b | a | a | a | a | a | b | a | a | a | b | b | b | b | a | b | a | a | a | a | a | a | a | b | b | a | a | a | a | a | b | a | b | b | b | b | a |
| 43 | a | b | a | a | a | a | b | a | a | a | a | b | a | a | a | a | a | a | a | b | a | a | b | b | b | a | b | a | a | a | a | a | a | a | b | b | a | a | a | a | a | a | b | a | a | a | a | a |
| 44 | a | b | a | a | a | a | b | a | a | a | a | b | a | a | a | a | a | a | a | b | a | a | b | b | b | a | b | a | a | a | a | a | a | a | b | b | a | a | a | a | a | a | b | a | a | a | a | a |
| 45 | a | b | a | a | a | a | b | a | a | a | a | b | a | a | a | a | a | a | a | b | a | a | b | b | b | a | b | a | a | a | a | a | a | a | b | b | a | a | a | a | a | a | b | a | a | a | a | a |
| 46 | a | b | a | a | a | a | b | b | a | a | a | b | a | a | a | a | a | b | a | a | a | b | b | b | b | a | b | b | a | b | a | a | a | a | b | b | a | a | a | a | a | a | b | a | a | a | a | a |
| 47 | a | b | a | a | a | a | a | a | a | a | a | b | a | a | a | a | a | a | a | b | a | b | b | b | b | a | b | a | a | a | a | a | a | a | a | a | a | a | a | a | a | a | a | a | a | a | a | b |
| 48 | b | a | a | a | a | a | b | a | a | a | a | b | a | a | a | a | a | a | a | b | a | a | b | b | b | a | b | a | a | a | a | a | a | a | b | b | a | a | a | a | a | a | a | a | a | a | a | b |
| 49 | a | b | a | a | a | a | b | a | a | a | a | b | a | a | a | a | a | b | a | a | a | b | b | b | b | a | b | a | a | a | a | a | a | a | b | b | a | a | a | a | a | a | c | b | b | b | b | a |
| 50 | a | b | a | a | a | a | b | a | a | a | a | b | a | a | a | a | a | b | a | a | a | b | b | b | b | a | b | a | a | a | a | a | a | a | b | b | a | a | a | a | a | a | c | b | b | b | b | a |
| 51 | b | a | b | b | a | b | a | b | a | a | a | a | b | b | b | a | b | a | a | b | b | b | b | a | b | a | a | a | a | a | a | a | a | a | b | b | b | b | b | b | b | a | a | a | a | a | a | a |
| 52 | c | b | b | b | a | b | b | b | a | a | a | b | a | a | b | a | b | a | a | b | d | a | b | b | a | a | a | b | a | b | a | a | a | a | b | b | b | b | a | a | a | a | b | a | a | a | a | a |
| 53 | b | a | b | b | a | b | a | b | a | a | a | a | b | b | b | a | b | a | a | b | d | a | b | b | a | a | a | b | a | b | b | b | a | b | a | a | b | b | b | b | b | b | a | b | b | b | b | a |
| 54 | b | a | b | b | a | b | a | b | a | a | a | a | b | b | b | a | b | a | a | b | d | a | b | b | b | b | a | b | a | b | b | b | a | b | a | a | b | b | b | b | b | b | a | b | b | b | b | a |

**Table S7** Continued

| **Accession** | ***AGPlarM1*** | ***AGPlarM2*** | ***AGPisoM2*** | ***AGPisoM3*** | ***AGPsma M2*** | ***AGPsma 1*** | ***GBSSⅡM1*** | ***SSI M1*** | ***SSI M2*** | ***SSI M3*** | ***SSIIc M1*** | ***SSIIc M2*** | ***SSIIc M3*** | ***SSIIc M4*** | ***SSIIb M1*** | ***SSIIb M2*** | ***SSIIb M3*** | ***SSIIa M1*** | ***SSIIa M2*** | ***SSIIa M3*** | ***SSIIIb M1*** | ***SSIIIb M2*** | ***SSIIIb M3*** | ***SSIIIb t1*** | ***SSIIIa M1*** | ***SSIIIa M2*** | ***SSIVa*** | ***SSIVb M1*** | ***SSIVb M2*** | ***SSIVb M3*** | ***SBE1 M1*** | ***SBE1 M2*** | ***SBE1 M3*** | ***SBE2 M4*** | ***SBEIIb M1*** | ***SBEIIb t1*** | ***SBEIIa M1*** | ***SBEIIa M2*** | ***ISA M1*** | ***ISA M2*** | ***ISA M3*** | ***PUL M1*** | ***PUL M2*** | ***PUL M3*** | ***PUL M4*** | ***PUL M5*** | ***PUL M6*** | ***PCR ACCI*** |
| --- | --- | --- | --- | --- | --- | --- | --- | --- | --- | --- | --- | --- | --- | --- | --- | --- | --- | --- | --- | --- | --- | --- | --- | --- | --- | --- | --- | --- | --- | --- | --- | --- | --- | --- | --- | --- | --- | --- | --- | --- | --- | --- | --- | --- | --- | --- | --- | --- |
| 55 | b | a | b | b | a | b | a | b | a | a | a | a | b | b | b | a | b | a | a | b | d | a | b | b | a | a | a | b | a | b | b | b | a | b | a | a | b | b | b | b | b | b | a | b | b | b | b | a |
| 56 | b | a | b | b | a | b | a | b | a | a | a | a | b | b | b | a | b | a | a | b | d | a | b | b | a | a | a | b | a | b | a | a | a | b | a | a | b | b | a | a | a | b | a | b | b | b | b | a |
| 57 | a | b | a | a | a | a | b | a | a | a | a | b | a | a | a | a | a | b | a | a | b | b | b | a | b | a | b | b | a | b | a | a | a | a | b | b | a | a | a | a | a | a | a | a | a | a | a | a |
| 58 | c | b | a | a | a | a | b | a | a | a | a | b | a | a | a | a | a | b | a | a | a | a | b | b | b | a | b | a | a | a | a | a | a | a | b | b | a | a | a | a | a | a | b | a | a | a | a | b |
| 59 | a | b | a | a | a | a | b | a | a | a | a | b | a | a | a | b | a | b | a | a | a | a | b | b | b | a | b | b | a | b | a | a | a | a | b | b | a | a | a | a | a | a | b | a | a | a | a | a |
| 60 | c | b | a | a | a | a | b | a | a | a | a | b | a | a | a | a | a | b | a | a | a | a | b | b | b | a | b | a | a | a | a | a | a | a | b | b | a | a | a | a | a | a | b | a | a | a | a | b |
| 61 | a | b | a | a | a | a | b | a | a | a | a | b | a | a | a | a | a | b | a | a | a | a | b | b | b | a | b | a | a | a | a | a | a | a | b | b | a | a | a | a | a | a | b | a | a | a | a | b |
| 62 | a | b | b | b | a | a | b | a | a | a | a | b | a | a | a | a | a | b | a | a | d | a | b | b | b | a | b | a | a | a | a | a | a | a | b | b | a | a | a | a | a | a | a | a | a | a | a | a |
| 63 | a | b | a | a | a | a | b | a | a | a | a | b | a | a | b | a | b | b | a | a | d | a | b | b | b | a | b | a | a | a | a | a | a | a | b | b | b | b | a | a | a | a | a | a | a | a | a | a |
| 64 | a | b | a | a | a | a | b | b | a | a | a | b | a | a | a | a | a | b | a | a | a | b | b | b | b | a | b | a | a | a | a | a | a | a | b | b | a | a | a | a | a | a | a | a | a | a | a | a |
| 65 | a | b | a | a | a | a | b | a | a | a | a | b | a | a | a | a | a | b | a | a | a | a | b | b | b | a | b | b | a | b | a | a | a | a | a | a | a | a | a | a | a | a | b | a | a | a | a | b |
| 66 | a | b | a | a | a | a | b | a | a | a | a | b | a | a | a | a | a | b | a | a | a | b | b | b | b | a | b | b | a | b | a | a | a | a | b | b | a | a | a | a | a | a | a | a | a | a | a | a |
| 67 | a | b | a | a | a | a | b | a | a | a | a | b | a | a | a | a | a | b | a | a | a | b | b | b | b | a | b | b | a | b | a | a | a | a | b | b | a | a | a | a | a | a | a | a | a | a | a | a |
| 68 | c | b | a | a | a | a | b | a | a | a | a | b | a | a | a | a | a | a | a | b | a | a | b | b | b | a | b | b | a | b | a | a | a | a | b | b | a | a | a | a | a | a | b | a | a | a | a | b |
| 69 | b | a | a | a | a | b | a | b | a | a | a | a | b | b | b | a | b | a | a | b | d | a | b | b | a | a | a | b | a | b | b | b | a | b | a | a | b | b | b | b | b | b | a | b | b | b | b | a |
| 70 | b | a | b | b | a | b | a | b | a | a | a | a | b | b | b | a | b | a | a | b | d | a | b | b | a | a | a | b | a | b | b | b | a | b | a | a | b | b | b | b | b | b | a | b | b | b | b | a |
| 71 | b | a | b | b | a | b | a | b | a | a | a | a | b | b | b | a | b | a | a | b | d | a | b | b | a | a | a | b | a | b | b | b | a | b | a | a | b | b | b | b | b | b | a | b | b | b | b | a |
| 72 | b | a | b | b | a | b | a | b | a | a | a | a | b | b | b | a | b | a | a | b | d | a | b | b | a | a | a | b | a | b | b | b | a | b | a | a | b | b | b | b | b | b | a | b | b | b | b | a |
| 73 | b | a | a | a | a | b | a | b | a | a | a | a | b | b | b | a | b | a | a | b | d | a | b | b | a | a | a | b | a | b | a | a | a | a | a | a | b | b | b | b | b | b | a | b | b | b | b | a |

a, b, c, d, e, f indicated the types of genetic polymorphisms in 73 rice accessions under specific molecular marker
